# Molecular-Genetic Basis of Pulmonary Arterial Hypertension (PAH)

**DOI:** 10.3390/cimb48060572

**Published:** 2026-05-29

**Authors:** Mark Okot, Aneesa Ahmed, Colin W. Wright, Md Talat Nasim

**Affiliations:** 1 Translational Medicine Laboratory, School of Pharmacy, Optometry and Medical Sciences, University of Bradford, Bradford BD7 1DP, UK; markokot71@gmail.com (M.O.); a.ahmed237@bradford.ac.uk (A.A.); c.w.wright@bradford.ac.uk (C.W.W.); 2 Kabarole District Local Government, Boma, Fort Portal P.O. Box 38, Uganda; 3 Conservation Through Public Health, Plot 3 Mapeera Lane, Uringi Crescent, Entebbe P.O. Box 75298, Uganda; 4 Institute of Health and Social Care, University of Bradford, Bradford BD7 1DP, UK; 5 Centre for Health Agricultural and Socio-Economic Advancements (CHASA), Lalmonirhat 5500, Bangladesh

**Keywords:** pulmonary arterial hypertension, BMPR2, heritable PAH, genetic predisposition, TGF-β signaling, SNPs, sequencing, translational medicine

## Abstract

Pulmonary arterial hypertension (PAH) is a progressive, fatal disease of the pulmonary vasculature characterized by obliterative remodeling of small pulmonary arteries, leading to sustained elevation of pulmonary vascular resistance, right ventricular failure, and premature death. The diagnostic gold standard remains right heart catheterization, requiring a mean pulmonary artery pressure greater than 20 mmHg at rest, a pulmonary arterial wedge pressure of 15 mmHg or below, and a pulmonary vascular resistance exceeding 2 Wood units. PAH is an autosomal dominant disorder with markedly incomplete penetrance of approximately 20–30%, indicating that germline mutations alone are insufficient to cause disease. Disease manifestation requires additional “second hits”, including chronic hypoxia, systemic inflammation, hemodynamic stress, hormonal influences, and common genetic modifiers such as single-nucleotide polymorphisms (SNPs). This genetic and environmental complexity underpins the broad clinical heterogeneity observed across PAH subtypes, which include idiopathic PAH, heritable PAH, and disease associated with connective tissue disorders, HIV infection, portal hypertension, congenital heart disease, schistosomiasis, and drug or toxin exposure. This review provides a comprehensive and critical appraisal of the molecular-genetic architecture of PAH. Thirty genes have now been implicated in disease pathogenesis, spanning seven functional categories: receptors of the TGF-β/BMP signaling family (*BMPR2*, *ACVRL1*, *ENG*, *BMPR1B*); circulating BMP ligands (*GDF2*, *BMP10*); transcription factors (*TBX4*, *SOX17*, *KLF4*, *FOXF1*, *SMAD1*, *SMAD4*, *SMAD9*); membrane and polyamine transporters (*ATP13A3*, *AQP1*); potassium channel regulators (*KCNA5*, *KCNK3*, *ABCC8*); metabolic and mitochondrial genes (*EIF2AK4*, *NFU1*, *GGCX*); signaling receptors and structural proteins (*NOTCH3*, *KDR*, *CAV1*, *PLEKHH2*); vasoactive and extracellular matrix regulators (*KLK1*, *CBLN2*, *CD248*); and epigenetic regulators (*TET2*, *TOPBP1*). Among these, *BMPR2* is the dominant contributor, accounting for 53–86% of heritable PAH and 14–35% of idiopathic cases. The remaining genes each account for fewer than 5% of cases individually, collectively reflecting a broad landscape of rare and ultra-rare genetic contributions. For each gene, we critically evaluate the strength of genetic evidence, pathogenic mechanisms, degree of mechanistic resolution, and clinical relevance. We further discuss the contribution of emerging technologies, including whole-genome sequencing, single-cell and spatial transcriptomics, multi-omics integration, iPSC-derived vascular models, and artificial intelligence, to expanding the PAH genetic architecture beyond single-gene discovery. A key theme across this landscape is convergence: despite mechanistic diversity at the gene level, most PAH-associated variants ultimately impair endothelial quiescence, promote smooth muscle proliferation, and drive apoptosis resistance through disruption of BMP signaling amplitude, transcriptional stability, ion channel homeostasis, metabolic integrity, or epigenetic regulation. This convergence supports both a unified therapeutic rationale and a precision medicine framework for genotype-stratified intervention in PAH.

## 1. Introduction

Pulmonary arterial hypertension (PAH) is a lethal, progressive disorder and a subtype of the broader condition of pulmonary hypertension. It is defined as a mean pulmonary artery pressure of >20 mmHg at rest, pulmonary arterial wedge pressure ≤ 15 mmHg, and pulmonary vascular resistance > 2 Wood units [1]. PAH is an incurable disorder estimated to affect 146,000 people across the EU, USA, and Japan [2]. Due to limited surveillance and healthcare access, the true prevalence is likely higher in developing countries. The condition is characterized by intima and media hyperplasia, formation of plexiform lesions in the pulmonary vasculature leading to elevated pulmonary arterial pressure, vascular resistance, and consequential right-heart failure. Symptoms of pulmonary arterial hypertension include difficulty in breathing, fatigue, weakness, syncope, angina, and edema [3].

Clinical classification of PAH

Idiopathic PAH

Heritable PAH

Drug- and toxin-induced PAH

PAH associated with:

Connective tissue disease

HIV infection

Portal hypertension

Congenital heart disease

Schistosomiasis

PAH in response to long-term calcium channel blocker use

PAH with overt features of venous/capillary involvement

Persistent PH of the newborn syndrome [4]

**Figure 1:**
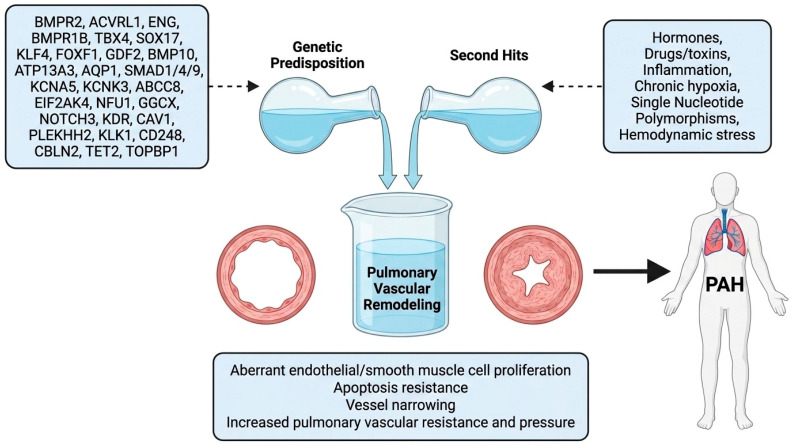
Genetic and environmental determinants of PAH. Schematic representation of the “two-hit” hypothesis in the pathogenesis of PAH. Genetic susceptibility arises from variants or mutations in genes associated with vascular remodeling and signaling pathways. Environmental or physiological “second hits” such as hormones, drugs and toxins, inflammation, chronic hypoxia, hemodynamic stress, and SNPs, further drive disease onset. The interaction of these factors leads to pulmonary vascular remodeling, a major pathological feature in PAH. The authors created this figure using FigureLabs.

All heritable PAH and some cases of idiopathic PAH are caused by dysfunctions in certain genes (Figure 1). According to [5], at least 5% of idiopathic PAH patients have a family history of the disease. The presence of mutations in these genes can trigger the condition, especially in the presence of a “second hit” such as chronic hypoxia, inflammation, hemodynamic stress, hormones, drugs, and single-nucleotide polymorphisms (SNPs) (Figure 1). A second hit refers to a secondary exposure that acts on a genetically predisposed background, initiating or accelerating the pathological processes (pulmonary vascular remodeling) that lead to the development of overt PAH. PAH is inherited in an autosomal dominant manner with an incomplete penetrance of around 20% [5]. This low penetrance, even in the presence of a mutation, corroborates the requirement for a “second hit” to manifest PAH in some cases. Sex (estrogen) plays a role in penetrance, evidenced by females having a 2.3 times higher preponderance of PAH than males [6]. Chronic hypoxia, hemodynamic stress, and inflammation cause pulmonary vascular injury, which leads to vascular remodeling, thereby precipitating PAH. Drugs such as methamphetamines, anorexigens, and amphetamines increase serotonin activity; serotonin is both a potent vasoconstrictor and a smooth muscle cell mitogen, promoting vascular remodeling. SNPs alter transcription efficiency of the *TGF-β1* gene, hence distorting the BMP/TGF-β balance by upregulating TGF-β signaling [7] (Table 1). Aberrant TGF-β signaling disrupts pulmonary vascular cell proliferation, survival, and migration, thereby causing vascular remodeling—a hallmark feature of PAH. Cigarette smoking represents a further environmental second hit of clinical relevance. Tobacco smoke constituents cause direct oxidative endothelial injury, promote pulmonary vascular inflammation, and impair nitric oxide-mediated vasodilation, thereby lowering the threshold for disease manifestation in genetically predisposed individuals. This is particularly pertinent in PAH cases associated with chronic obstructive pulmonary disease (COPD) or combined pulmonary fibrosis and emphysema, where smoking-induced vascular injury acts synergistically with genetic susceptibility to accelerate pulmonary vascular remodeling [8].

This review discusses all genes currently implicated in PAH pathogenesis (Figure 1). These genes have been grouped according to function: receptors of the TGF-β family (*BMPR2*, *ACVRL1*, *ENG*, and *BMPR1B*), ligands of the BMP signaling pathway (*GDF2* and *BMP10*), transcription factors (*TBX4*, *SOX17*, *KLF4*, *FOXF1*, and *SMAD1*/*4/8*), membrane transporters (*ATP13A3* and *AQP1*), potassium ion transporters (*KCNA5*, *KCNK3*, and *ABCC8*), metabolic genes (*EIF2AK4*, *NFU-1*, and *GGCX*), other signaling receptors (*NOTCH3* and *KDR*), membrane/structural proteins (*CAV-1* and *PLEKHH2*), vasoactive regulation (*KLK1*), extracellular/matrix-related (*CD248* and *CBLN2*), and epigenetic/nuclear regulation (*TET2* and *TOPBP1*).

It should be noted that the depth of coverage across gene sections in this review reflects the available published literature. Genes such as *BMPR2*, *ACVRL1*, *GDF2*, *SOX17*, and *EIF2AK4* are discussed in greater detail because they are supported by extensive genetic, functional, and clinical data accumulated over two decades. By contrast, genes such as *PLEKHH2*, *KLK1*, *CBLN2*, *CD248*, *TOPBP1*, *NFU1*, and *GGCX* are covered more concisely because the published evidence base remains limited, consisting largely of small cohort reports, single functional studies, or preliminary genetic associations. Where evidence is sparse, this review explicitly acknowledges that limitation rather than over-interpreting available data.

## 2. Methods

A literature search was conducted using Google Scholar to identify studies reporting mutations of genes implicated in pulmonary arterial hypertension (PAH) in patient cohorts. Search terms comprised the names and symbols of individual PAH-associated genes combined with the terms “pulmonary arterial hypertension”, “mutation”, “variant”, and “pathogenesis”. Retrieved articles were assessed for relevance and quality, with priority given to primary genetic studies, large cohort analyses, and high-quality secondary reviews. No formal date restriction was applied; however, emphasis was placed on studies published from 2000 onwards, corresponding to the discovery of *BMPR2* as the first causal gene in heritable PAH. Evidence quality was evaluated using the ClinGen Pulmonary Hypertension Gene Curation Expert Panel framework, and genes are categorized throughout this review accordingly.

## 3. Genes Implicated in PAH Pathogenesis

### 3.1. Receptors of the TGF-β Family

**Figure 2:**
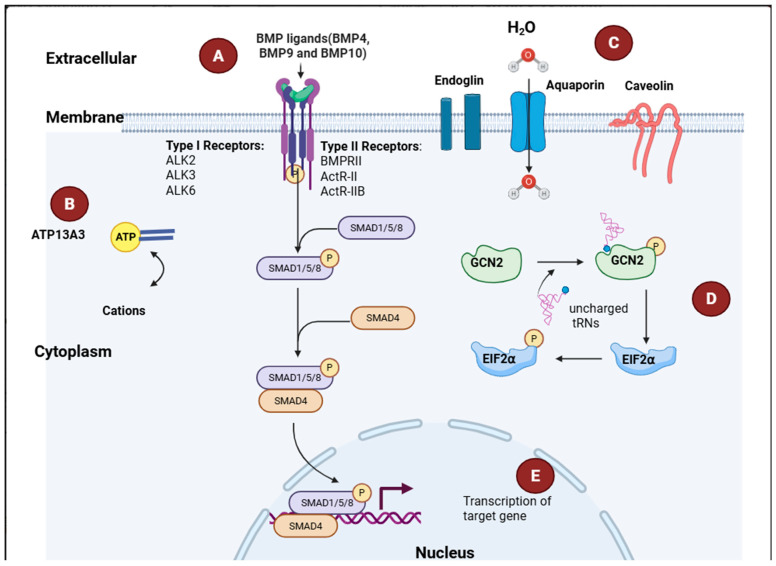
Schematic diagram depicting the signaling pathways and genes implicated in pulmonary arterial hypertension (PAH). **Ⓐ** BMP/*BMPR2* signaling: Ligand binding (*BMP4*, *BMP9*, and *BMP10*) activates Type II receptors, which phosphorylate Type I receptor and accessory receptor Endoglin to form a complex. This complex leads to SMAD signaling and transcription of genes (e.g., *ID1*) that are essential for maintaining vascular homeostasis. Impairment contributes to endothelial dysfunction. **Ⓑ** *ATP13A3*: Involved in polyamine transport across the cell membrane. **Ⓒ** Aquaporin/Caveolin: Modulates water transport and endothelial signaling; mutations alter vascular tone and trigger remodeling. **Ⓓ** GCN2-EIF2α integrated stress response: Detects amino acid deprivation through uncharged tRNAs; prolonged activation drives vascular remodeling. **Ⓔ** Alteration of target gene transcription. Alterations in transcription may lead to excessive cell proliferation, resistance to apoptosis, and abnormal migration and differentiation. The authors created this figure using FigureLabs.

The TGF-β family comprises a diverse group of cytokines such as bone morphogenetic proteins (BMPs), activins, growth differentiation factors (GDFs), nodal, and inhibins [9] that modulate key cellular processes such as proliferation, differentiation, apoptosis, and extracellular matrix formation. TGF-β ligands signal through a receptor system composed of type I, type II, and type III co-receptors. In the BMP signaling pathway, the type II (BMPRII, ActRIIA, and ActRIIB)-type I receptor (*ALK1*, *ALK2*, *ALK3*, *ALK6*) complex activates SMAD1/5/8 (Figure 2). In the TGF-β signaling pathway, the type II (TGFβRII, ActRIIA, ActRIIB)-type I receptor (*ALK4*, *ALK5*, *ALK7*) complex activates SMAD2/3, which leads to downstream transcription of genes such as *PAI-1* that are responsible for the cellular changes (aberrant proliferation and apoptosis resistance) seen in PAH [9]. Type III receptors (endoglin and betaglycan) function as auxiliary receptors, facilitating ligand presentation to type II–type I receptor complexes.

#### 3.1.1. *BMPR2*

The bone morphogenetic protein receptor type 2 (*BMPR2*), a member of the transforming growth factor-β (TGF-β) superfamily, plays a central role in regulating vascular cell proliferation, differentiation, and apoptosis. The *BMPR2* gene, located on chromosome 2q33, encodes a receptor comprising an extracellular ligand-binding domain (exons 1–3), a transmembrane domain (exon 4), a serine/threonine kinase domain (exons 5–11), and a cytoplasmic C-terminal domain (exons 12–13) [10,11].

*BMPR2* was first identified as a causal gene for heritable pulmonary arterial hypertension (PAH) in 2000 through independent studies demonstrating heterozygous germline mutations in affected families [12,13,14]. According to the ClinGen Pulmonary Hypertension Gene Curation Expert Panel, *BMPR2* is classified as a definitive gene for PAH, representing the most robust and reproducible genetic association in the disease [15].

Pathogenic *BMPR2* variants are highly heterogeneous, including nonsense, frameshift, splice-site mutations, and large genomic rearrangements, most of which result in haploinsufficiency through nonsense-mediated decay or truncated protein products [16,17,18]. These variants account for approximately 53–86% of heritable PAH cases and 14–35% of idiopathic PAH [19]. However, despite this strong association, BMPR2 mutations exhibit incomplete penetrance of approximately 20–30% [20], indicating that mutation carriage alone is insufficient to drive disease development.

This incomplete penetrance represents a key area of uncertainty and suggests that additional genetic modifiers, epigenetic mechanisms, and environmental exposures contribute to disease expression. Variability in penetrance and disease severity among carriers highlights substantial heterogeneity that is not fully explained by current genetic models, underscoring limitations in our understanding of disease initiation.

At the molecular level, *BMPR2* mediates signaling through both canonical and non-canonical pathways. Canonical signaling involves ligand binding (particularly *BMP9* and *BMP10*), receptor complex formation, and phosphorylation of *SMAD1*/*5/8* (Figure 2), which regulate transcription of genes essential for maintaining vascular quiescence [21,22]. Disruption of this pathway leads to impaired anti-proliferative signaling in endothelial cells.

In parallel, *BMPR2* dysfunction is associated with activation of non-canonical signaling pathways, including ERK, p38 MAPK, JNK, and PI3K/AKT cascades, which contribute to endothelial dysfunction, apoptosis resistance, and vascular remodeling [22]. However, the relative contribution of these pathways to disease initiation versus progression remains incompletely defined, reflecting a broader limitation in mechanistic resolution across PAH studies.

A central pathogenic feature of PAH is the imbalance between BMP and TGF-β signaling. Reduced *BMPR2*-mediated signaling, coupled with relatively enhanced TGF-β pathway activity, promotes a pro-proliferative and anti-apoptotic vascular phenotype. Similar pathway dysregulation is observed in other PAH-associated genes within the BMP signaling axis, including *ACVRL1*, *ENG*, *SMAD9*, and *GDF2*. Compared with these genes, *BMPR2* has the most extensive genetic and functional evidence supporting causality, whereas several others demonstrate more variable or context-dependent associations [15]. This distinction underscores the importance of differentiating between definitive causal genes and those with emerging or more limited evidence.

Clinically, *BMPR2* mutation carriers tend to present at a younger age, exhibit more severe hemodynamic impairment, and have poorer survival compared with non-carriers [8]. However, reduced *BMPR2* protein expression has also been observed in PAH patients without identifiable *BMPR2* mutations, suggesting that *BMPR2* downregulation may reflect both primary genetic effects and secondary disease-associated processes [23]. This observation complicates direct attribution of causality and highlights the interplay between genetic predisposition and disease progression.

Experimental animal models have confirmed the importance of *BMPR2* in vascular development and signaling [24]; however, these models incompletely recapitulate the chronic, heterogeneous, and incompletely penetrant nature of human PAH. While these systems provide important mechanistic insights, their translational relevance remains limited, particularly in modeling late-onset or environmentally influenced disease.

Taken together, *BMPR2* represents the most well-established genetic contributor to PAH, but its incomplete penetrance, variable expressivity, and interaction with broader signaling networks emphasize that it functions within a complex, multifactorial disease framework rather than as a singular deterministic cause.

#### 3.1.2. *ACVRL1*

The *ACVRL1* gene encodes activin receptor-like kinase 1 (*ALK1*), a type I serine/threonine kinase receptor within the transforming growth factor-β (TGF-β) superfamily that is predominantly expressed in endothelial cells. Located on chromosome 12q13, *ALK1* forms part of a heteromeric receptor complex that mediates signaling in response to circulating ligands such as *BMP9* and *BMP10* (Figure 2), thereby promoting endothelial quiescence and vascular stability [25,26].

Pathogenic variants in *ACVRL1* were first identified in patients with hereditary hemorrhagic telangiectasia (HHT), establishing its role in vascular dysplasia [27,28]. Subsequent studies demonstrated that heterozygous *ACVRL1* mutations are also associated with pulmonary arterial hypertension (PAH), particularly in the context of HHT-associated PAH [29,30]. According to the ClinGen Pulmonary Hypertension Gene Curation Expert Panel, *ACVRL1* is classified as a definitive PAH gene, although it contributes less frequently to disease compared with *BMPR2* [15].

The mutation spectrum of *ACVRL1* includes missense, nonsense, frameshift, and splice-site variants [31], many of which affect functionally critical regions such as the kinase domain. These mutations account for approximately 3–5% of heritable PAH cases and a smaller proportion of idiopathic PAH, underscoring their relatively modest contribution compared with *BMPR2*-associated disease [30]. Clinically, *ACVRL1* mutation carriers tend to present at a younger age and may exhibit more severe disease, particularly in individuals with overlapping HHT features, suggesting a phenotype shaped by combined vascular dysplasia and pulmonary vascular remodeling [30].

At the signaling level, *ALK1* is a key mediator of endothelial BMP signaling. Ligand binding promotes phosphorylation of *SMAD1*/*5/8* and downstream transcriptional programs that maintain vascular homeostasis. Loss of *ACVRL1* function attenuates this pathway, reducing endothelial quiescence and increasing susceptibility to pathological remodeling [25,26]. In parallel, diminished *ALK1* activity has been associated with relative enhancement of TGF-β signaling, shifting endothelial cells toward pro-proliferative and anti-apoptotic states. However, much of this mechanistic framework is derived from in vitro systems and animal models, and direct confirmation in human pulmonary vascular tissue remains limited, constraining definitive causal interpretation.

Comparison with *BMPR2*-related PAH highlights both shared and distinct features within the BMP/TGF-β signaling axis. While both genes converge on impaired SMAD-mediated signaling, *BMPR2* mutations are more prevalent and better characterized in isolated PAH, whereas *ACVRL1*-associated disease is frequently embedded within a broader syndromic vascular context. This distinction suggests that perturbations at different nodes of the same pathway may confer varying degrees of penetrance and tissue specificity, rather than equivalent pathogenic effects.

Several unresolved questions remain. The relative contribution of endothelial versus smooth muscle dysfunction, the extent to which *ALK1* signaling defects initiate disease versus amplify existing vascular injury, and the marked variability in clinical expression among mutation carriers all point to incomplete mechanistic resolution. These uncertainties support a model in which *ACVRL1* mutations create a sensitized endothelial state that requires additional genetic or environmental factors to produce overt PAH.

This endothelial signaling vulnerability is further exemplified by co-receptors such as *ENG*, which function alongside *ALK1* within the same receptor complex and provide complementary insight into the role of disrupted BMP signaling in PAH pathogenesis.

#### 3.1.3. *ENDOGLIN*

Endoglin (*ENG*) is a transmembrane glycoprotein predominantly expressed in endothelial cells, where it functions as a co-receptor within the transforming growth factor-β (TGF-β)/bone morphogenetic protein (BMP) signaling pathway. Located on chromosome 9q34, *ENG* encodes a protein belonging to the zona pellucida family, characterized by a conserved extracellular domain that facilitates ligand binding and stabilization of receptor complexes [32,33]. Through its interaction with type I receptors such as *ALK1* and type II receptors, endoglin modulates endothelial responses to circulating ligands, particularly *BMP9* and *BMP10* (Figure 2) [26].

Pathogenic variants in *ENG* were identified in patients with hereditary hemorrhagic telangiectasia (HHT), establishing its role in vascular dysplasia [27,32]. Subsequent studies have demonstrated that *ENG* mutations are also associated with pulmonary arterial hypertension (PAH), particularly in the context of HHT-associated disease [34]. According to the ClinGen Pulmonary Hypertension Gene Curation Expert Panel, *ENG* is classified as a definitive PAH gene, although its contribution is less frequent than *BMPR2* and is often observed within a syndromic vascular context [15].

Endoglin enhances BMP signaling by facilitating ligand–receptor complex formation and promoting activation of the *SMAD1*/*5/8* pathway, thereby supporting endothelial quiescence and vascular integrity. Loss of *ENG* disrupts this signaling axis and alters endothelial proliferation, migration, and apoptosis. Experimental models demonstrate reduced BMP pathway activity, evidenced by decreased *SMAD1*/*5/8* phosphorylation in *ENG*-deficient systems [35]. This signaling impairment closely parallels that observed in *BMPR2*- and *ACVRL1*-deficient models, indicating convergence on a shared downstream pathway rather than gene-specific effects.

Despite this mechanistic alignment, several limitations constrain interpretation. Much of the available evidence derives from murine models and in vitro systems, which do not fully capture the structural and hemodynamic complexity of the human pulmonary vasculature. Consequently, it remains unclear whether *ENG* deficiency directly initiates pulmonary vascular disease or instead amplifies remodeling processes in an already compromised vascular environment. This distinction is particularly relevant given that ENG-associated PAH frequently occurs in the setting of systemic vascular abnormalities such as HHT.

Clinically, *ENG* mutation carriers, similar to those with *ACVRL1* mutations, tend to present with earlier disease onset and may experience more severe outcomes, including reduced survival [34]. However, available cohort sizes are limited, and marked phenotypic variability suggests the influence of additional genetic or environmental modifiers.

In comparison with *BMPR2*, *ENG* contributes less frequently to PAH and is more commonly associated with syndromic vascular disease, underscoring differences in penetrance and disease context despite shared pathway involvement. This contrast supports a model in which disruption of endothelial BMP signaling represents a central pathogenic axis, while the specific clinical phenotype depends on the level and context of pathway perturbation.

Extending this pathway-level framework, circulating ligands such as *GDF2* (*BMP9*) provide further insight into how upstream modulation of BMP signaling influences disease susceptibility.

#### 3.1.4. *BMPR1B*

Bone morphogenetic protein receptor type 1B (*BMPR1B*), also known as *ALK6*, is a type I serine/threonine kinase receptor within the transforming growth factor-β (TGF-β) superfamily that participates in canonical BMP signaling. Structurally, *BMPR1B* comprises an extracellular ligand-binding domain, a glycine–serine (GS) domain required for receptor activation, a transmembrane domain, and an intracellular kinase domain (Table 2) [36]. While these structural features are conserved across BMP type I receptors, *BMPR1B* appears to have a more restricted role in pulmonary vascular biology, with comparatively lower endothelial expression than receptors such as *BMPR2* and *ACVRL1*.

Evidence linking *BMPR1B* to pulmonary arterial hypertension (PAH) remains limited. Initial sequencing studies identified two missense variants (S160N and F392L) in pediatric idiopathic PAH patients [37]. Functional assessment demonstrated that the F392L variant, located within the kinase domain, more substantially altered receptor signaling than the S160N variant, consistent with observations in *BMPR2* where kinase-domain mutations exert greater functional impact. However, unlike canonical loss-of-function mutations in *BMPR2* or *ACVRL1*, both *BMPR1B* variants retained the capacity to induce *SMAD1*/5/8 signaling in vitro, raising uncertainty regarding whether these alterations represent partial loss, gain, or context-dependent modulation of receptor activity.

This ambiguity highlights an important distinction between *BMPR1B* and established PAH genes within the BMP pathway. In contrast to *BMPR2*, *ACVRL1*, and *ENG*, where convergent evidence supports reduced BMP signaling as a central pathogenic mechanism, *BMPR1B*-associated variants do not consistently demonstrate impaired downstream signaling. This lack of directional consistency weakens causal inference and suggests that *BMPR1B* may not operate as a primary driver of pathway insufficiency, but rather as a context-dependent modulator of signaling output.

Consistent with this interpretation, *BMPR1B* is not currently classified as a PAH-associated gene by the Clinical Genome Resource Pulmonary Hypertension Gene Curation Expert Panel, which designates the gene as having limited evidence [15]. This classification reflects the small number of reported variants, the absence of replication in independent cohorts, and limited in vivo functional validation.

From a mechanistic perspective, *BMPR1B* variants may influence receptor complex composition or ligand responsiveness, thereby subtly altering SMAD signaling dynamics rather than producing the marked signaling deficits observed in *BMPR2*-related disease. This contrasts with ligand-level defects (e.g., *BMP9*/*BMP10*) or receptor haploinsufficiency, where reduced signaling amplitude is more clearly established. In this context, *BMPR1B* is better positioned alongside emerging or modifier genes, where effects on pathway tuning rather than pathway disruption may contribute to disease susceptibility.

A key unresolved issue is whether *BMPR1B* variants exert measurable effects under physiological conditions, where receptor stoichiometry, ligand gradients, and hemodynamic forces differ substantially from in vitro systems. The restriction of reported variants to small pediatric cohorts further limits generalizability and prevents robust genotype–phenotype correlation.

Current evidence does not support a definitive pathogenic role for *BMPR1B* in PAH. Instead, the gene represents a biologically plausible but genetically under-validated component of the BMP signaling network, illustrating the importance of distinguishing between core pathway drivers and peripheral modulators when interpreting PAH genetics.

### 3.2. Ligands of the BMP Signaling Pathway

BMPs are a subclass of the TGF-β superfamily that specifically signal through type I receptors *ACVRL1* (*ALK1*), ACVR1 (*ALK2*), BMPR1A (*ALK3*), and *BMPR1B* (*ALK6*), consequently leading to the phosphorylation of *SMAD1*/*5/8* (Figure 2) [38]. BMPs such as GDF5, GDF6, GDF7, *BMP2*, *BMP4*, BMP5, BMP6, BMP7, BMP8A, BMP8B, *BMP9*, *BMP10*, and BMP15 modulate several cellular processes, including proliferation, growth, differentiation, and apoptosis. Mutations in genes encoding these ligands disrupt the BMP/TGF-β signaling balance, triggering the vascular remodeling changes characteristic of PAH.

#### 3.2.1. *GDF2*

The *GDF2* gene encodes bone morphogenetic protein 9 (*BMP9*), a circulating ligand within the transforming growth factor-β (TGF-β) superfamily that plays a central role in maintaining vascular homeostasis (Table 2). *BMP9* is synthesized predominantly in the liver and circulates in an active form, signaling through receptor complexes composed of *BMPR2* and type I receptors such as *ACVRL1* on endothelial cells [25]. Through activation of canonical *SMAD1*/*5/8* signaling, *BMP9* promotes endothelial quiescence and suppresses inappropriate vascular proliferation.

In contrast to receptor-level mutations (e.g., *BMPR2*, *ACVRL1*, *ENG*), *GDF2* variants disrupt BMP signaling at the level of ligand availability, demonstrating that pulmonary arterial hypertension (PAH) can arise from impaired signal input as well as defective signal transduction. Sequencing studies first identified pathogenic *GDF2* variants in PAH cohorts, with functional analyses showing reduced circulating *BMP9* levels and diminished downstream SMAD activation [39,40]. Consistent with this, the Clinical Genome Resource Pulmonary Hypertension Gene Curation Expert Panel classifies *GDF2* as a definitive PAH gene [15].

Most *GDF2* mutations result in reduced secretion or stability of *BMP9*, leading to decreased activation of *BMPR2*/*ALK1* receptor complexes. The downstream consequence is attenuation of *SMAD1*/*5/8* signaling and a relative shift toward TGF-β–dominant pathways, promoting endothelial dysfunction, apoptosis resistance, and vascular remodeling. This signaling imbalance closely mirrors that observed in receptor-level defects, indicating that diverse genetic lesions converge on a shared pathway-level deficiency rather than producing gene-specific phenotypes.

A key mechanistic insight from *GDF2*-associated PAH is the apparent importance of ligand dosage. Biallelic loss-of-function mutations are associated with early-onset and severe disease, whereas heterozygous carriers demonstrate variable penetrance and later onset [39,41]. This dosage effect parallels observations in *BMPR2* haploinsufficiency and supports a threshold model in which partial reduction in BMP signaling lowers endothelial resilience without being independently sufficient to cause disease.

However, this framework is complicated by the presence of functional redundancy within the BMP ligand axis. *BMP9* shares substantial overlap with *BMP10*, which can also activate *ALK1*-mediated signaling. This raises an unresolved question: whether *GDF2* mutations cause disease primarily through absolute reduction in signaling, or through disruption of the relative balance between circulating BMP ligands. The partial preservation of signaling in some mutation carriers suggests that compensatory mechanisms may exist, but these are not sufficient to fully prevent disease under stress conditions.

Despite strong genetic and biochemical evidence, several limitations remain. Much of the current understanding is derived from circulating *BMP9* measurements and in vitro endothelial assays, which may not fully reflect ligand gradients, receptor distribution, or hemodynamic influences present in the pulmonary circulation. Furthermore, incomplete penetrance in heterozygous carriers indicates that *GDF2* deficiency alone is often insufficient to initiate PAH, supporting a multi-hit model involving additional genetic, inflammatory, or environmental modifiers.

Compared with receptor-associated genes, *GDF2* highlights the importance of upstream signal availability in maintaining endothelial homeostasis. Together with defects in receptors (*BMPR2*, *ACVRL1*, *ENG*) and downstream mediators (SMADs), these findings support a unified model in which PAH arises from quantitative and context-dependent disruption of BMP signaling across multiple levels of the pathway, rather than from isolated gene-specific mechanisms.

#### 3.2.2. *BMP10*

The *BMP10* gene encodes bone morphogenetic protein 10, a circulating ligand within the transforming growth factor-β (TGF-β) superfamily (Figure 2) that plays a critical role in cardiovascular development, particularly in cardiac morphogenesis and ventricular trabeculation during embryogenesis. In adults, *BMP10* is predominantly produced in the right atrium and circulates as an endocrine-like vascular ligand [42,43]. *BMP10* signals through type II BMP receptors in complex with type I receptors such as *ACVRL1* (*ALK1*), activating canonical *SMAD1*/*5/8* signaling and downstream transcriptional programs that promote endothelial quiescence and vascular stability [25].

*BMP10* exhibits substantial functional overlap with *BMP9* (encoded by *GDF2*), with both ligands acting as high-affinity activators of *ALK1*-mediated signaling in endothelial cells. However, an important distinction lies in their physiological roles: *BMP9* is the dominant circulating ligand under basal conditions, whereas *BMP10* appears to contribute more selectively, with expression enriched in cardiac tissue. This asymmetry suggests that *BMP10* may function as a context-dependent or compensatory ligand rather than a primary regulator of endothelial homeostasis.

From a pathobiological perspective, disruption of *BMP10* signaling would be expected to impair SMAD-dependent pathways and promote endothelial dysfunction, similar to defects observed in *GDF2* or receptor-level mutations. However, direct genetic evidence linking *BMP10* variants to pulmonary arterial hypertension (PAH) remains limited. Unlike *GDF2*, which is classified as a definitive PAH gene, *BMP10* has not yet achieved equivalent evidentiary support within frameworks such as the Clinical Genome Resource Pulmonary Hypertension Gene Curation Expert Panel [15]. This distinction is critical, as it places *BMP10* within the category of biologically plausible but genetically under-validated contributors.

A central unresolved issue is the extent to which *BMP10* contributes independently to disease versus acting within a redundant ligand network. Functional overlap with *BMP9* raises the possibility that isolated *BMP10* deficiency may be buffered by preserved *BMP9* signaling, thereby limiting phenotypic expression. Conversely, combined or cumulative reductions in BMP ligand availability could reduce overall signaling below a critical threshold, predisposing to disease. This “ligand dosage” model aligns with observations in *GDF2* and *BMPR2*-associated PAH, where partial signaling deficits confer susceptibility rather than deterministic causality.

Emerging studies also suggest that *BMP10* may exert effects beyond canonical SMAD signaling. Experimental data indicate that altered *BMP10* activity can modulate inflammatory pathways, including upregulation of chemokines such as *CCL2*, potentially promoting monocyte recruitment and vascular inflammation [44]. While this introduces an additional mechanistic dimension linking BMP signaling to immune-mediated remodeling, these findings remain preliminary and are largely derived from in vitro systems.

Several limitations constrain current interpretation. The majority of mechanistic data originate from developmental biology and experimental models, which may not accurately reflect adult pulmonary vascular disease. The absence of well-characterized human mutation cohorts limits genotype–phenotype correlation and prevents clear attribution of causality. These gaps contrast with the robust genetic and functional evidence supporting *BMP9* (*GDF2*), *BMPR2*, and *ACVRL1*, highlighting a gradient of evidence within the BMP signaling axis.

Therefore, *BMP10* is best conceptualized as a complementary ligand within the BMP signaling network that contributes to endothelial homeostasis but has not yet been definitively implicated in PAH. Its role likely reflects modulation of overall ligand availability rather than a primary pathogenic driver, reinforcing the broader concept that quantitative and context-dependent disruption of BMP signaling, rather than single-gene defects, underlies pulmonary vascular disease.

### 3.3. Transcription Factors

Transcription factors play a crucial role in the pathogenesis of PAH by regulating the expression of genes that drive endothelial dysfunction, PASMC proliferation, metabolic reprogramming, inflammation, and extracellular matrix remodeling. Abnormal activation, suppression, or mutation of transcription factors contributes to the pro-proliferative, anti-apoptotic, and vasoconstrictive phenotype characteristic of PAH.

#### 3.3.1. *TBX4*

Mutations in the *TBX4* gene have emerged as an important genetic contributor to childhood-onset pulmonary arterial hypertension (PAH). Early cohort studies demonstrated that a substantial proportion of pediatric PAH cases harbor either microdeletions at chromosome 17q23.2 encompassing *TBX4* or pathogenic sequence variants within the gene [45]. In contrast, *TBX4* variants are identified in only a small fraction of adult PAH cases (approximately 1–2%), indicating a marked age-dependent penetrance and supporting a distinct developmental contribution to disease susceptibility.

*TBX4* encodes a T-box transcription factor that plays a central role in embryonic limb and pulmonary development. A key downstream target is fibroblast growth factor 10 (*FGF10*), which is essential for lung branching morphogenesis and pulmonary vascular formation [46]. Disruption of the *TBX4*–*FGF10* axis therefore provides a biologically plausible mechanism for early-life vulnerability, whereby abnormal lung and vascular development results in a structurally compromised pulmonary vascular bed that is less capable of adapting to postnatal hemodynamic demands.

This developmental framework distinguishes *TBX4* from canonical PAH genes such as *BMPR2*, *ACVRL1*, and *GDF2*, which primarily perturb endothelial signaling pathways in established vasculature. Instead, *TBX4*-associated PAH is best conceptualized as a developmentally primed condition, in which impaired formation of the pulmonary vascular architecture lowers the threshold for later disease expression rather than directly initiating pathogenic signaling in adulthood.

However, this model remains partially inferential. While developmental defects are well supported by experimental and genetic data, the direct molecular link between *TBX4* disruption and the characteristic vascular remodeling seen in PAH—namely smooth muscle proliferation, endothelial dysfunction, and occlusive lesion formation—has not been fully delineated in human systems. Most mechanistic insights derive from developmental biology and animal models, which may not fully recapitulate the postnatal pulmonary vascular environment.

Genotype–phenotype relationships further complicate interpretation. *TBX4* variants are associated with a broad phenotypic spectrum, ranging from isolated PAH to small patella syndrome and other skeletal abnormalities, indicating pleiotropic effects of gene disruption. This variability, together with incomplete penetrance, suggests that *TBX4* mutations alone are often insufficient to cause disease and that additional genetic modifiers or environmental stressors are required for clinical manifestation. Such observations are consistent with a multi-hit model of PAH pathogenesis.

From an evidence standpoint, *TBX4* is classified as a definitive PAH gene by the Clinical Genome Resource Pulmonary Hypertension Gene Curation Expert Panel [15], supported by reproducible genetic associations and consistent clinical phenotypes, particularly in pediatric populations. This classification reflects strong genetic evidence rather than complete mechanistic resolution, highlighting a gap between genotype identification and pathway-level understanding.

In comparison with BMP pathway genes, *TBX4* occupies a distinct position within the PAH genetic landscape. Whereas BMP-related genes converge on dysregulated endothelial signaling, *TBX4* reflects disruption of early developmental programming. This distinction underscores the biological heterogeneity of PAH and supports the concept that disease can arise from both structural predisposition and signaling imbalance, which may ultimately converge on shared downstream pathways of vascular remodeling.

*TBX4* represents a key determinant of pediatric PAH characterized by developmental dysregulation, variable penetrance, and mechanistic divergence from classical signaling-driven forms of disease. Studies integrating human pulmonary tissue analysis, developmental modeling, and longitudinal clinical data are needed to clarify how early developmental defects translate into progressive vascular pathology.

#### 3.3.2. *SOX17*

The *SOX17* gene, located on chromosome 8q11.23, encodes the SRY-box transcription factor 17, a key regulator of endothelial development, arterial specification, and vascular homeostasis. *SOX17* functions as a lineage-defining transcription factor that maintains endothelial identity, particularly in arterial endothelial cells, by coordinating developmental and postnatal gene expression programs involved in vascular stability [47]. The gene consists of a single coding exon (exon 2), encoding the conserved high-mobility group (HMG) DNA-binding domain and a C-terminal region involved in protein–protein interactions, including β-catenin binding.

Pathogenic *SOX17* variants identified in pulmonary arterial hypertension (PAH) cohorts include missense mutations affecting the HMG-box domain, as well as truncating variants such as nonsense and frameshift mutations [48]. These variants are predicted to impair DNA binding and disrupt transcriptional regulation of endothelial gene networks. According to the Clinical Genome Resource Pulmonary Hypertension Gene Curation Expert Panel, *SOX17* is classified as a definitive PAH gene [15], reflecting replicated genetic association and consistent functional evidence across independent cohorts.

*SOX17* regulates endothelial signaling networks that intersect with both Wnt/β-catenin and Notch pathways. Loss-of-function studies demonstrate derepression of β-catenin-dependent transcriptional programs and impaired maintenance of endothelial quiescence, contributing to proliferative and migratory phenotypes in vascular endothelium [47]. Additional experimental evidence suggests modulation of hepatocyte growth factor (HGF)/c-Met signaling, although this remains primarily based on cellular models and has not been fully validated in human pulmonary vascular tissue [49]. These data support a model in which *SOX17* does not initiate proliferative signaling directly but rather destabilizes transcriptional control of multiple downstream pathways that govern endothelial behavior.

A key mechanistic feature of *SOX17*-associated PAH is its strong context dependence. Endothelial-specific *SOX17* loss is insufficient to induce pulmonary arterial hypertension under baseline conditions, whereas disease phenotypes emerge in the presence of additional stressors such as chronic hypoxia or vascular injury [49]. This provides experimental support for a multi-hit model in which *SOX17* deficiency lowers endothelial resilience but requires secondary environmental or physiological triggers for full disease expression. This behavior closely parallels that observed in *BMPR2* and *GDF2*-associated PAH, despite differences in molecular level of action.

From a comparative standpoint, *SOX17* occupies a distinct mechanistic position relative to BMP pathway genes such as *BMPR2*, *ACVRL1*, *ENG*, and *GDF2*. Whereas these genes regulate extracellular ligand–receptor signaling within the TGF-β superfamily, *SOX17* functions downstream as an intracellular transcriptional integrator of endothelial identity. Despite this hierarchical difference, both classes converge on a shared endpoint of impaired endothelial quiescence and maladaptive vascular remodeling, suggesting pathway convergence rather than pathway exclusivity in PAH pathogenesis.

Clinically, *SOX17* mutations account for approximately 3% of PAH cases, with variable penetrance and expressivity across carriers [50]. This variability further supports a non-deterministic model of disease in which *SOX17* variants act as susceptibility alleles rather than sole causal drivers, with phenotypic expression shaped by genetic modifiers and environmental exposures.

*SOX17* represents a definitive PAH gene that contributes to disease through disruption of endothelial transcriptional stability rather than extracellular signaling. However, key mechanistic gaps remain, particularly in defining how *SOX17*-dependent transcriptional programs interface with BMP signaling and inflammatory or hypoxic stress pathways in human pulmonary vascular tissue.

#### 3.3.3. *KLF4*

Kruppel-like factor 4 (*KLF4*), located on chromosome 9q31, encodes a zinc finger transcription factor of the Kruppel-like family, characterized by three C-terminal C2H2 zinc finger domains that mediate DNA binding. In vascular biology, *KLF4* is highly expressed in endothelial cells, where it contributes to the maintenance of endothelial identity and adaptive responses to biomechanical and inflammatory stress [51] (Table 2). Unlike monogenic pulmonary arterial hypertension (PAH) genes, *KLF4* is not currently classified as a disease-causing locus but is increasingly recognized as a context-dependent regulator of endothelial quiescence.

At the transcriptional level, *KLF4* regulates multiple endothelial effector pathways, including nitric oxide signaling via endothelial nitric oxide synthase (eNOS), prostacyclin synthesis, and endothelin signaling. In PAH lung tissue, reduced *KLF4* expression has been associated with downregulation of eNOS, endothelin receptor subtype B (ETB), and prostacyclin synthase, alongside increased endothelin-1 expression [52]. This coordinated shift reflects a reprogramming of endothelial signaling toward vasoconstriction, inflammation, and smooth muscle activation, which are central features of pulmonary vascular remodeling.

*KLF4* functions less as a pathway-specific signaling component and more as a transcriptional integration node that stabilizes endothelial quiescence under shear stress and inflammatory stimulation. Its downregulation therefore does not initiate a discrete signaling defect but instead lowers the transcriptional threshold for activation of vasoconstrictive and proliferative programs. However, the current evidence base is largely derived from human tissue expression profiling and in vitro endothelial models, limiting causal inference regarding whether *KLF4* loss is primary or secondary to established vascular injury.

From a genetic perspective, *KLF4* differs fundamentally from established PAH genes such as *SOX17*, *BMPR2*, or *TBX4*, which harbor recurrent germline pathogenic variants with demonstrable disease segregation. In contrast, no reproducible monogenic *KLF4* variant spectrum has been established in PAH, and its contribution is not currently supported at the level of a definitive or moderate ClinGen gene–disease relationship. This positions *KLF4* outside the core heritable PAH gene set and instead within a broader category of transcriptional susceptibility factors.

*KLF4* aligns more closely with endothelial state regulators such as *CAV1* or *FOXF1*, which modulate vascular phenotype stability without acting as primary signaling initiators. In this framework, *KLF4* loss is best interpreted as a permissive alteration that amplifies downstream consequences of established pathogenic pathways, including BMP/TGF-β dysregulation, inflammatory activation, and hypoxia-driven remodeling.

Several limitations constrain current interpretation. First, most available data is correlative, and reduced *KLF4* expression may represent a downstream consequence of endothelial injury rather than a primary event. Second, in vivo models of endothelial-specific *KLF4* loss in pulmonary hypertension remain limited and context-dependent, preventing definitive assignment of causality. Third, the absence of validated pathogenic germline variants further limits its classification as a monogenic PAH gene.

*KLF4* is best conceptualized as a transcriptional gatekeeper of endothelial stability whose downregulation facilitates, rather than initiates, the pathological vascular phenotype in PAH. Its role is therefore most consistent with a disease modifier that integrates biomechanical and inflammatory stress into transcriptional programs that converge on vascular remodeling.

#### 3.3.4. *FOXF1*

*FOXF1* (Forkhead box F1), located on chromosome 16p24.1, encodes a forkhead family transcription factor characterized by a conserved winged-helix DNA-binding domain. During embryogenesis, *FOXF1* is a critical regulator of mesenchymal–endothelial crosstalk and is required for proper pulmonary vascular and alveolar capillary network development [53] (Table 2). In contrast, its role in adult pulmonary vascular homeostasis is less well defined, although emerging evidence supports a continued contribution to endothelial maintenance and injury responses.

Functional studies in pulmonary endothelial systems suggest that *FOXF1* contributes to endothelial stability by regulating angiogenic competence and DNA damage response pathways. In pulmonary artery endothelial cells derived from pulmonary arterial hypertension (PAH) patients, *FOXF1* overexpression has been associated with increased angiogenic signaling, including upregulation of *VEGFR2* and *CLDN5*, alongside enhanced expression of DNA repair-related mediators such as TP53 and ATM [54]. Conversely, *FOXF1* suppression in endothelial models impairs migration, reduces angiogenic capacity, and disrupts DNA damage response signaling, indicating a role in maintaining endothelial adaptability under stress rather than directly controlling proliferative signaling.

This evidence positions *FOXF1* as a regulator of endothelial repair capacity and genomic stability rather than a primary determinant of vascular tone or canonical proliferative signaling pathways. Its function appears to center on preserving endothelial resilience following injury, particularly under conditions of oxidative stress or impaired vascular regeneration. However, the current evidence base is largely derived from in vitro systems, and the extent to which these mechanisms operate in vivo within the human pulmonary circulation remains uncertain.

From a genetic standpoint, *FOXF1* differs from definitive PAH genes such as *BMPR2*, *SOX17*, and *ACVRL1*, which exhibit recurrent pathogenic variants with established disease segregation. In contrast, *FOXF1* has not been consistently implicated in monogenic PAH, and its contribution is not currently established as a definitive gene–disease relationship. This supports its interpretation as a susceptibility or modifier locus rather than a primary causal driver in most PAH contexts.

*FOXF1* occupies a distinct mechanistic position within PAH biology. Unlike *SOX17*, which directly regulates endothelial transcriptional programs linked to vascular identity, or *KLF4*, which integrates inflammatory and biomechanical cues, *FOXF1* primarily influences endothelial repair and genomic maintenance pathways. This positions it within a complementary axis of vascular resilience, functioning alongside, but mechanistically distinct from, core BMP/TGF-β signaling components and ion-channel or metabolic regulators.

Key limitations persist. Most mechanistic evidence derives from endothelial cell culture models, which do not fully replicate the multicellular and hemodynamic complexity of pulmonary vascular remodeling. Second, definitive human genetic evidence linking *FOXF1* variation to heritable PAH is limited, and its role may be context-dependent rather than deterministic. Third, the upstream and downstream integration of *FOXF1* with established PAH pathways, including BMP signaling and inflammatory cascades, remains incompletely defined.

Therefore, *FOXF1* is best conceptualized as a transcriptional regulator of endothelial repair and genomic integrity that modulates susceptibility to pulmonary vascular injury. Rather than initiating PAH, *FOXF1* dysfunction likely lowers endothelial resilience, thereby amplifying disease progression in the presence of primary pathogenic insults.

#### 3.3.5. *SMADs* *1*, *4*, and *8*

*SMAD1* is a receptor-regulated SMAD (R-SMAD) that functions as a key intracellular effector of bone morphogenetic protein (BMP) signaling. Following ligand binding to BMP receptor complexes, type II receptors phosphorylate type I receptors, which in turn phosphorylate *SMAD1*. Activated *SMAD1* then forms a complex with the common mediator *SMAD4*, translocates to the nucleus, and regulates transcription of downstream target genes involved in endothelial homeostasis, proliferation, and vascular stability (Figure 2). In this context, *SMAD1* serves as a central intracellular signal transducer that converts extracellular BMP signaling intensity into transcriptional responses within pulmonary vascular cells.

Genetic studies have identified rare variants in SMAD pathway genes in pulmonary arterial hypertension (PAH) cohorts, including *SMAD1* missense variants (e.g., c.8T>C; p.V3A) and splice-site alterations, as well as *SMAD4* variants, which are associated with impaired BMP signaling activity [55]. These findings provide important genetic evidence that disruption at the level of intracellular signal transduction can phenocopy upstream defects in BMP receptors and ligands. However, these variants are extremely rare, and robust genotype–phenotype correlations remain limited, preventing definitive conclusions regarding their overall contribution to PAH susceptibility.

*SMAD8* (also known as *SMAD9*) is another receptor-regulated SMAD that participates in BMP signaling but exhibits distinct functional properties compared with *SMAD1*/*5*. Although *SMAD8* can bind DNA and participate in transcriptional complexes, it demonstrates weaker transcriptional activation capacity and context-dependent regulatory effects, partly due to structural differences in its linker region [56]. These differences allow *SMAD8* to modulate BMP-driven transcriptional output in a manner that can attenuate or fine-tune signaling responses depending on cellular context.

Alterations in *SMAD8* have been proposed to disrupt endothelial BMP signaling by interfering with the balance of *SMAD1*/*5*-mediated transcriptional programs. One potential mechanism is competition for receptor-mediated phosphorylation or *SMAD4* binding, which may reduce the effective transcriptional output of canonical BMP signaling. Alternatively, altered *SMAD8* structure may shift transcriptional complexes toward a less active or dysregulated state, thereby contributing to endothelial dysfunction and vascular remodeling. However, these mechanistic models remain largely based on in vitro experimental systems and have not been fully validated in human pulmonary vascular tissue.

Why do alterations in a relatively weak transcriptional effector such as *SMAD8* produce meaningful biological effects in the BMP pathway? One possibility is that even modest perturbations in SMAD complex composition can significantly alter transcriptional programs in endothelial cells, given the sensitivity of BMP signaling to dosage and stoichiometric balance. Another possibility is that *SMAD8* variants exert dominant-negative effects under specific cellular conditions, thereby amplifying downstream signaling disruption.

Despite this, the overall evidence base remains limited, and in vivo models are needed to clarify whether *SMAD1*/*SMAD4* dysfunction and *SMAD8* dysregulation converge on shared pathogenic pathways or represent distinct mechanisms of endothelial injury. In particular, future studies should address whether SMAD-level perturbations act primarily as upstream amplifiers of BMP signaling defects or as independent modifiers of transcriptional endothelial states within the pulmonary circulation.

### 3.4. Membrane Transporters

Membrane transporters are integral proteins that regulate the movement of ions, water, metabolites, lipids, and signaling molecules across the cell membrane, thereby controlling essential processes such as osmosis, nutrient uptake, intercellular communication, and mitochondrial function. These proteins are either channels or carriers/pumps. Channels allow molecules to move across the cell membrane by passive diffusion driven by electrochemical gradients, whereas carriers/pumps rely on either primary (ATP hydrolysis) or secondary active transporters.

#### 3.4.1. *ATP13A3*

ATP13A3 encodes a transmembrane P5-type ATPase localized predominantly to recycling endosomes, where it plays a key role in polyamine transport and intracellular polyamine homeostasis. Polyamines are essential regulators of cellular proliferation, survival, and cytoskeletal organization, and are particularly important for maintaining endothelial integrity and vascular barrier function (Figure 2) (Table 2). In this context, *ATP13A3* is increasingly recognized as a metabolic regulator of endothelial fitness rather than a classical signaling molecule within the transforming growth factor-β (TGF-β)/bone morphogenetic protein (BMP) pathway [57].

Pathogenic variants in *ATP13A3*, including biallelic missense and loss-of-function mutations, have been identified in pediatric and early-onset pulmonary arterial hypertension (PAH), suggesting that reduced gene dosage is associated with more severe disease phenotypes [57,58]. Functional studies indicate that *ATP13A3* deficiency impairs polyamine uptake, leading to reduced endothelial cell proliferation, increased apoptosis, and disruption of monolayer integrity in pulmonary artery endothelial cells. These alterations are consistent with a primary defect in endothelial survival rather than direct induction of a proliferative phenotype.

*ATP13A3* dysfunction has been shown to compromise endothelial junctional stability, resulting in increased vascular permeability, including enhanced susceptibility to thrombin-induced barrier disruption. This loss of barrier integrity provides a plausible link between metabolic dysregulation and early endothelial injury in PAH. These effects converge with pathways implicated in other endothelial stability genes such as *AQP1*, *CAV1*, and *SOX17*, suggesting that impaired barrier maintenance represents a shared pathogenic axis across genetically diverse forms of PAH.

A paradox arises: that *ATP13A3* deficiency reduces endothelial proliferation in vitro, whereas PAH is characterized by pathological vascular remodeling and proliferative vascular lesions. This discrepancy suggests that *ATP13A3* is unlikely to function as a direct pro-proliferative driver. Instead, current evidence supports a model in which *ATP13A3* loss primarily induces endothelial injury and metabolic stress, thereby creating a permissive environment for secondary proliferative responses in adjacent smooth muscle cells and fibroblasts. In this framework, vascular remodeling is interpreted as a compensatory and maladaptive response to primary endothelial dysfunction rather than a direct consequence of *ATP13A3*-driven proliferation.

According to the ClinGen Pulmonary Hypertension Gene Curation Expert Panel, *ATP13A3* is classified as a definitive PAH gene, supporting a reproducible association between loss-of-function variants and disease across multiple cohorts [15]. However, despite this genetic strength, key mechanistic gaps remain. In particular, the absence of endothelial-specific *ATP13A3* knockout models and limited in vivo human functional data restricts understanding of how polyamine dysregulation translates into pulmonary vascular remodeling over time.

*ATP13A3* represents a distinct class of PAH-associated genes that act through metabolic and membrane transport dysfunction rather than canonical signaling pathways. Its role in disease is best understood as a contributor to endothelial vulnerability and barrier failure, which synergizes with other genetic and environmental insults to initiate and amplify pulmonary vascular injury.

#### 3.4.2. *AQP1*

Aquaporin 1 (*AQP1*), encoded by the *AQP1* gene, is a transmembrane water channel widely expressed in endothelial cells, where it facilitates rapid, bidirectional water transport across the plasma membrane. Structural and functional studies established that *AQP1* forms tetrameric channels that selectively permit water flux in response to osmotic gradients, thereby regulating [59,60] (Figure 2) (Table 2). Beyond passive transport, *AQP1* has been implicated in endothelial cell migration through localized water influx at the leading edge, which supports lamellipodia formation and cytoskeletal remodeling [61].

In pulmonary arterial hypertension (PAH), altered *AQP1* expression has been reported in pulmonary vascular cells. Experimental studies demonstrated that *AQP1* overexpression enhances endothelial and pulmonary artery smooth muscle cell proliferation and migration, whereas *AQP1* inhibition attenuates these responses [62]. In parallel, functional assays have shown that *AQP1* increases endothelial monolayer permeability and reduces barrier resistance, consistent with disruption of cell–cell junction integrity. These findings suggest that *AQP1* influences vascular remodeling not through classical receptor-mediated signaling, but by modulating biophysical properties of the endothelial barrier.

Despite these observations, the direction of causality remains unresolved. Increased *AQP1* expression may represent an adaptive response to hypoxia, shear stress, or endothelial injury rather than a primary pathogenic trigger. This interpretation is supported by the limited availability of longitudinal human data and the predominance of in vitro experimental systems, which do not fully recapitulate pulmonary vascular hemodynamics.

Genetic analyses have identified rare variants in *AQP1* among PAH patients, including the recurrent missense variant c.376C>T (p.Arg126Cys) [63]. However, direct functional validation linking these variants to altered channel activity or endothelial dysfunction in vivo remains limited. Consistent with current evidence-based classifications, *AQP1* is not considered a definitive PAH gene by the Clinical Genome Resource and is best categorized as an emerging or candidate gene with incomplete mechanistic validation [15].

*AQP1*-associated phenotypes appear to reflect dysregulated water flux and altered cell volume dynamics, which can influence endothelial migration, barrier permeability, and responsiveness to mechanical stress. However, these effects are likely context-dependent and may require coexisting environmental or molecular “second hits” to contribute meaningfully to disease progression.

Comparison with *ATP13A3* highlights a notable convergence in pathogenic outcome despite divergent upstream mechanisms. Whereas *ATP13A3* deficiency impairs polyamine transport, leading to reduced endothelial proliferation and increased apoptosis, *AQP1* upregulation has been associated with enhanced proliferative and migratory behavior. Despite these opposing cellular phenotypes, both pathways converge on endothelial barrier dysfunction, supporting the concept that barrier failure represents a common downstream event in PAH pathogenesis.

In summary, *AQP1* is best conceptualized as a modulator of endothelial barrier dynamics whose dysregulation may contribute to pulmonary vascular remodeling indirectly, rather than functioning as a primary driver of disease. Further in vivo studies, particularly those integrating hemodynamic stress models and human endothelial systems, will be required to define its precise role within the broader PAH genetic and mechanistic landscape.

### 3.5. Potassium Ion Transporters

Potassium ion transporters, including voltage-gated, ATP-sensitive, and two-pore potassium channels, are membrane proteins that modulate the movement of potassium ions across the cell membrane, thereby playing a pivotal role in regulating membrane potential, vascular tone, and smooth muscle cell proliferation.

#### 3.5.1. *KCNA5*

**Figure 3:**
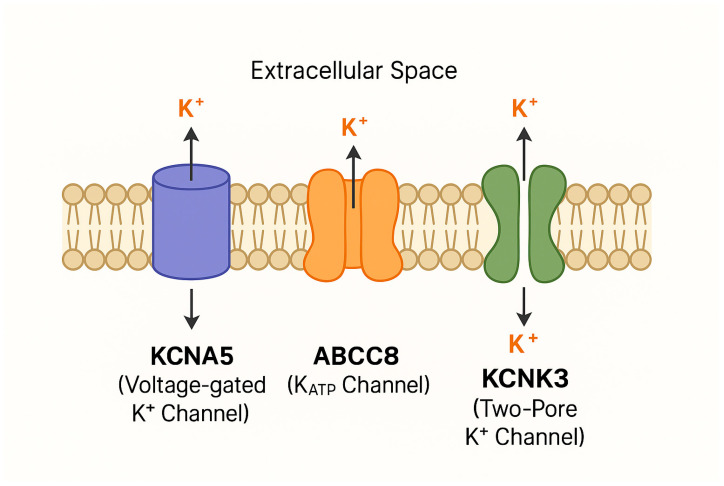
Genes involved in potassium ion transport across the cell membrane contribute to PAH. *KCNA5* controls the function of the voltage-gated K^+^ channel (Kv1.5). *ABCC8* regulates ATP-sensitive potassium channel function. *KCNK3* modulates the function of the PH-sensitive potassium channels. Mutations of these genes lead to membrane depolarization that, in turn, leads to vasoconstriction of the pulmonary artery.

The *KCNA5* gene, located on chromosome 12p13, encodes the Kv1.5 channel alpha subunit, a voltage-gated potassium (K^+^) channel that is a key determinant of resting membrane potential in pulmonary artery smooth muscle cells (Figure 3). Unlike multi-exon ion channel genes, *KCNA5* contains a single exon, which limits alternative splicing and makes functional variants more likely to directly disrupt channel structure. Kv1.5 plays an important role in maintaining pulmonary vascular tone through modulation of calcium ions (Ca^2+^) influx across voltage-dependent Ca^2+^ channels (VDCC) (Figure 3). This places *KCNA5* within a distinct smooth muscle-centric pathogenic axis, in contrast to endothelial-focused genes such as *SOX17*, *ATP13A3*, and *AQP1*.

Under physiological conditions, Kv1.5 activity maintains a hyperpolarized membrane state, limiting calcium entry and thereby preventing excessive vasoconstriction. Loss or inhibition of Kv1.5 function leads to membrane depolarization, increased calcium influx, and enhanced PASMC contraction, resulting in sustained vasoconstriction. In addition, Kv1.5 dysfunction has been linked to impaired apoptosis through reduced apoptotic volume decrease and altered caspase activation, promoting PASMC survival and contributing to vascular remodeling [64,65]. Together, these effects, vasoconstriction and apoptosis resistance, recapitulate core pathological features of pulmonary arterial hypertension (PAH) and align mechanistically with other ion channel-associated PAH genes such as *KCNK3* and *ABCC8*.

*KCNA5* encodes a channel protein with a relatively simple gene structure compared with multi-exon regulatory genes, meaning that missense variants are more likely to have direct functional consequences on channel gating, conductance, or membrane expression. Rare *KCNA5* variants, including Arg184Pro and Gly384Arg, have been identified in PAH cohorts and shown to impair channel function, reduce Kv1.5 current density, and promote a pro-proliferative, anti-apoptotic PASMC phenotype [65]. Although these variants account for a small proportion of PAH cases, their consistent electrophysiological effects provide strong mechanistic support for the role of membrane depolarization and ion handling abnormalities in disease pathogenesis.

However, several important limitations must be acknowledged. Much of the mechanistic evidence is derived from in vitro electrophysiological studies and experimental modulation of channel expression, which may not fully replicate the physiological effects of endogenous pathogenic variants in human pulmonary vasculature. In addition, the rarity of *KCNA5* variants and limited replication across independent cohorts constrain robust genotype–phenotype correlations, making it difficult to determine their true population-level contribution to PAH.

From a pathogenic standpoint, *KCNA5* is best understood as a moderate-effect susceptibility gene rather than a high-penetrance monogenic driver. Its variants likely reduce electrical and apoptotic reserve in PASMCs, thereby increasing vulnerability to secondary insults such as hypoxia, inflammation, or upstream genetic defects (e.g., *BMPR2* dysfunction). This positions *KCNA5* within a broader framework of ion channel dysregulation in PAH, where impaired membrane excitability contributes to sustained vasoconstriction and vascular remodeling but typically requires additional “second hits” for full disease expression.

In conclusion, *KCNA5* dysfunction highlights the importance of bioelectric signaling in pulmonary vascular homeostasis and reinforces the concept that PAH pathogenesis arises from the convergence of endothelial, smooth muscle, and metabolic abnormalities across multiple biological layers.

#### 3.5.2. *KCNK3*

Whole-exome sequencing studies have identified pathogenic variants in *KCNK3*, which encodes the pH-sensitive two-pore domain potassium channel TASK-1. These variants account for approximately 1–3% of pulmonary arterial hypertension (PAH) cases, establishing *KCNK3* as one of the earliest identified ion channel–associated PAH genes [66] (Figure 3).

TASK-1 is widely expressed across multiple tissues, including pulmonary artery smooth muscle cells (PASMCs), cardiac atria, right ventricle, adrenal gland, endothelium, and pancreas, where it contributes to the regulation of resting membrane potential. Unlike voltage-gated potassium channels such as Kv1.5 (*KCNA5*), TASK-1 functions as a constitutively active “leak” channel that stabilizes membrane potential independent of voltage-dependent activation. This continuous background activity makes TASK-1 particularly important for maintaining basal electrical stability in pulmonary vascular cells and preventing inappropriate depolarization.

Loss-of-function mutations in *KCNK3* reduce potassium efflux, leading to membrane depolarization and secondary activation of voltage-dependent calcium channels. The resulting increase in intracellular calcium promotes sustained pulmonary artery smooth muscle cell contraction and vasoconstriction, thereby contributing to elevated pulmonary vascular resistance and increased mean pulmonary arterial pressure [66]. Because TASK-1 operates continuously under physiological conditions, its dysfunction produces persistent rather than episodic electrical instability, resulting in chronic vasoconstrictive tone.

In addition to effects on vascular tone, *KCNK3* deficiency promotes vascular remodeling through enhanced PASMC proliferation and resistance to apoptosis. This dual phenotype, vasoconstriction combined with proliferative remodeling, closely mirrors that observed in other ion channel-associated PAH genes such as *KCNA5* and *ABCC8*, reinforcing the concept that disrupted potassium homeostasis represents a shared mechanistic class in PAH pathogenesis.

Despite strong mechanistic plausibility supported by electrophysiological and experimental studies, several limitations remain. Notably, penetrance among *KCNK3* mutation carriers is variable, indicating that channel dysfunction alone is insufficient to cause disease in all individuals. This variability supports a multi-hit model in which *KCNK3* loss reduces electrophysiological reserve and increases susceptibility to secondary stressors such as hypoxia, inflammation, metabolic dysfunction, or additional genetic variants.

Based on the ClinGen Pulmonary Hypertension Gene Curation Expert Panel, *KCNK3* is regarded as a definitive genetic contributor to PAH [15]. Its pathogenic role is most appropriately understood within a broader framework of ion channel dysregulation in PAH, where impaired potassium conductance contributes to sustained depolarization, calcium overload, and maladaptive vascular remodeling in conjunction with other genetic and environmental factors.

#### 3.5.3. *ABCC8*

The ATP-binding cassette sub-family C member 8 (*ABCC8*) gene encodes the sulfonylurea receptor 1 (SUR1), a regulatory subunit of ATP-sensitive potassium (KATP) channels. SUR1 is a key metabolic sensor that links intracellular energy status—particularly ATP/ADP ratios—to potassium channel activity, thereby coupling cellular metabolism to membrane excitability. In the pulmonary vasculature, KATP channels are important determinants of resting membrane potential and vascular tone, placing *ABCC8* at the intersection of metabolic regulation and electrophysiological control of vascular function (Figure 3) (Table 2).

Loss-of-function variants in *ABCC8* impair SUR1 function, resulting in reduced KATP channel activity and diminished potassium efflux [67]. Consistent with other potassium channelopathies implicated in pulmonary arterial hypertension (PAH), reduced KATP activity leads to membrane depolarization, increased opening of voltage-dependent calcium channels, and subsequent intracellular calcium overload. This promotes sustained pulmonary artery smooth muscle cell contraction, contributing to increased pulmonary vascular resistance and vasoconstriction. In parallel, *ABCC8* dysfunction has also been associated with increased proliferation and reduced apoptosis in both endothelial cells and smooth muscle cells, thereby contributing to vascular remodeling [68].

Unlike *KCNA5* (voltage-gated Kv1.5) and *KCNK3* (TASK-1 background leak channel), which regulate membrane potential primarily through electrical conductance properties, *ABCC8* uniquely integrates metabolic status with electrophysiological signaling. By gating channel activity in response to ATP availability, SUR1 links cellular energetic state to vascular tone regulation. This metabolic–electrical coupling is particularly relevant in PAH, a disease increasingly recognized as involving mitochondrial dysfunction, altered glycolytic flux, and metabolic reprogramming of vascular cells. In this framework, *ABCC8* represents a mechanistic bridge between metabolic stress and ion channel dysfunction, contributing to sustained depolarization under conditions of cellular energy imbalance.

Despite strong mechanistic plausibility supported by experimental models, several limitations remain. Most evidence for *ABCC8*-associated vascular dysfunction is derived from in vitro systems or heterologous expression models, which may not fully recapitulate the complex metabolic and hemodynamic environment of the human pulmonary circulation. The frequency, penetrance, and spectrum of *ABCC8* variants in large, well-characterized PAH cohorts remain incompletely defined, limiting precise genotype–phenotype correlations.

*ABCC8* is best conceptualized as a metabolic regulator of ion channel function whose dysfunction contributes to PAH through impaired ATP-sensitive potassium channel activity, leading to membrane depolarization, calcium overload, and downstream vascular remodeling. Rather than acting as an isolated driver, ABCC8 fits within a broader class of ion channel–metabolic convergence genes that amplify susceptibility to pulmonary vascular disease in the presence of additional genetic or environmental stressors.

### 3.6. Metabolic Genes

Although PAH has long been associated with dysfunctional vascular signaling pathways (most notably the BMP/TGF-β pathway), recent genetic studies have identified a group of genes, such as *EIF2AK4*, *NFU1*, and *GGCX*, that link PAH to disturbances in cellular metabolism, mitochondrial function, and protein modification. Mutations of these genes lead to metabolic stress responses that trigger pulmonary vascular remodeling.

#### 3.6.1. *EIF2AK4*

The Eukaryotic Translation Initiation Factor 2 Alpha Kinase 4 (*EIF2AK4*) gene encodes General Control Nonderepressible 2 (GCN2), a serine/threonine kinase that functions as a central regulator of the integrated stress response (ISR). GCN2 is activated by accumulation of uncharged transfer RNAs during amino acid deprivation and other cellular stress conditions. Once activated, it phosphorylates the α-subunit of eukaryotic initiation factor 2 (eIF2α), leading to global attenuation of cap-dependent protein translation while selectively permitting translation of stress-adaptive transcripts [69]. This adaptive translational reprogramming is essential for maintaining endothelial cell survival under hypoxic, inflammatory, and metabolic stress conditions characteristic of the pulmonary vascular microenvironment in pulmonary hypertension (Figure 2) (Table 2).

According to ClinGen curation, *EIF2AK4* is classified as a definitive gene for pulmonary veno-occlusive disease and pulmonary capillary hemangiomatosis, conditions within the pulmonary hypertension spectrum that can clinically overlap with PAH [15]. Biallelic loss-of-function mutations in *EIF2AK4* account for a significant proportion of heritable PVOD/PCH cases and have also been reported in a subset of PAH cohorts [70]. Although early studies established a predominantly autosomal recessive inheritance pattern [70,71], subsequent reports suggest that *EIF2AK4* variants may also modify disease severity in the presence of additional genetic lesions, particularly *BMPR2* mutations [72]. In such contexts, splice-site or truncating variants affecting functional domains of GCN2 can impair ribosomal association and stress response activation, thereby lowering the threshold for disease manifestation and accelerating disease progression.

*EIF2AK4* dysfunction disrupts the integrated stress response, resulting in impaired translational control during periods of cellular stress. Loss of appropriate eIF2α phosphorylation compromises the ability of endothelial and smooth muscle cells to adapt to hypoxia, oxidative stress, and inflammatory signaling. This leads to cellular vulnerability, maladaptive protein synthesis, and accumulation of proteotoxic stress, which can indirectly amplify dysregulation of key vascular signaling pathways, including BMP and TGF-β cascades.

*EIF2AK4* occupies a distinct mechanistic position compared with metabolic regulators such as *ABCC8* and *ATP13A3*. Whereas *ABCC8* primarily couples ATP availability to membrane excitability and *ATP13A3* regulates polyamine-dependent cellular metabolism, *EIF2AK4* governs cellular stress tolerance through translational control. This places *EIF2AK4* as a key determinant of endothelial resilience rather than a direct effector of proliferative or electrophysiological signaling.

Despite strong genetic evidence linking *EIF2AK4* loss-of-function to PVOD/PCH, several important limitations remain. Functional studies in human pulmonary vascular tissue are limited, making it difficult to determine whether *EIF2AK4* mutations act as primary initiators of vascular remodeling or as modifiers that exacerbate pre-existing injury. Furthermore, the molecular basis underlying the phenotypic divergence between PVOD/PCH and PAH in *EIF2AK4*-deficient states remains incompletely understood, despite shared vascular stress signatures. This suggests that *EIF2AK4* mutations define a stress-sensitized vascular phenotype, whose clinical expression is shaped by genetic background, environmental exposure, and co-existing molecular abnormalities within the pulmonary circulation.

#### 3.6.2. *NFU1*

*NFU1* encodes a mitochondrial iron–sulfur (Fe–S) cluster assembly factor required for the maturation and delivery of Fe–S cofactors to multiple mitochondrial enzymes, including components of the electron transport chain (notably complex II) and lipoic acid-dependent enzymes (Table 2). Fe–S clusters are essential prosthetic groups involved in electron transfer within respiratory complexes I–III, thereby supporting oxidative phosphorylation, mitochondrial ATP production, and redox homeostasis [73]. *NFU1* functions as a core component of mitochondrial biosynthetic machinery required for cellular energetic stability.

Pathogenic biallelic *NFU1* variants are classically associated with severe early-onset mitochondrial disease, and pulmonary hypertension has been reported as part of this systemic phenotype in a subset of cases. Functional studies of specific variants (e.g., G208C) suggest impaired Fe–S cluster transfer with downstream disruption of respiratory chain activity and a compensatory shift toward glycolytic metabolism [74]. While this metabolic reprogramming resembles the glycolytic phenotype observed in pulmonary arterial hypertension (PAH), current evidence does not establish *NFU1* as a recurrent monogenic cause of isolated PAH in large cohorts. Instead, its contribution appears to be strongest in multisystem mitochondrial disease contexts where pulmonary vascular disease may emerge as part of broader metabolic failure.

In addition to impaired oxidative phosphorylation, *NFU1* dysfunction is associated with increased mitochondrial reactive oxygen species (ROS) production and reduced antioxidant buffering capacity. These changes may promote endothelial injury and impair adaptive signaling.

When compared with *EIF2AK4*, which regulates adaptive translational control during cellular stress, *NFU1* operates further upstream by affecting mitochondrial bioenergetic capacity itself. *EIF2AK4*-related disease represents impaired stress adaptation, whereas *NFU1*-related pathology reflects impaired energy generation capacity. In both cases, pulmonary vascular involvement likely arises secondarily from reduced cellular resilience rather than pathway-specific vascular signaling defects typical of established PAH genes (e.g., *BMPR2*, *SOX17*, or *ATP13A3*).

Current evidence supporting a direct role for *NFU1* in PAH is limited and largely derived from case reports and small series. No large-scale genotype–phenotype studies have established its prevalence in idiopathic or heritable PAH cohorts, and disease specificity remains uncertain given its established role in systemic mitochondrial disorders. Accordingly, *NFU1* is best interpreted as a candidate metabolic gene associated with pulmonary vascular disease in the context of mitochondrial syndromes, rather than a definitively established PAH gene. Its precise role in isolated PAH remains to be defined, and further systematic genetic and functional studies are required to clarify its position within the pulmonary hypertension gene landscape.

#### 3.6.3. *GGCX*

The *GGCX* gene encodes γ-glutamyl carboxylase, an integral endoplasmic reticulum membrane enzyme responsible for vitamin K-dependent post-translational γ-carboxylation of glutamate residues to γ-carboxyglutamate (Gla) (Table 2). This modification is essential for the functional activity of multiple extracellular proteins involved in coagulation and vascular biology, including matrix Gla protein (*MGP*), which has been implicated in vascular calcification and growth factor regulation [75,76]. *GGCX* represents a post-translational regulatory node linking vitamin K metabolism to extracellular modulation of vascular signaling pathways.

The relevance of *GGCX* to pulmonary vascular disease is primarily mediated through its role in activating *MGP*. Fully γ-carboxylated *MGP* acts as an extracellular inhibitor of bone morphogenetic protein (BMP) signaling by binding BMP ligands (notably *BMP2* and *BMP4*) and limiting their interaction with BMP receptors, thereby modulating local signaling intensity within the vascular extracellular matrix [77]. In contrast, undercarboxylated (inactive) *MGP* loses this inhibitory capacity, which may result in altered extracellular BMP ligand bioavailability and dysregulated spatial control of signaling. However, the net functional consequence in pulmonary vascular tissue is not fully resolved and may differ depending on local ligand abundance, vascular bed, and disease stage.

Rare variants in *GGCX* have been reported in small pulmonary arterial hypertension (PAH) cohorts, with an estimated frequency of ~1% in selected genetic screens [75]. However, in a ClinGen-consistent framework, the current evidence base remains limited, as replication across large independent PAH cohorts and robust segregation or functional validation data are still lacking. Accordingly, *GGCX* should not yet be considered a definitive PAH disease gene.

From a mechanistic standpoint, *GGCX*-related perturbation introduces a distinct regulatory layer in the BMP pathway that differs fundamentally from canonical PAH genes such as *BMPR2*, *ACVRL1*, or SMADs, which directly alter intracellular signaling. Instead, *GGCX* acts indirectly by modulating extracellular BMP ligand regulation through *MGP* activity. This places it within an emerging category of extracellular matrix-mediated signaling modulators, rather than classical receptor–ligand–transducer defects.

A key conceptual complexity arises from the fact that impaired *MGP* carboxylation may increase extracellular BMP ligand availability, potentially enhancing BMP signaling. This contrasts with the reduced BMP signaling typically observed in *BMPR2*- or *GDF2*-associated PAH. Experimental studies in vascular calcification biology suggest that *MGP* function is context-dependent and may differentially regulate BMP signaling intensity and localization rather than acting as a simple on/off inhibitor [77]. Consequently, whether altered *GGCX* activity results in net BMP pathway activation or dysregulated signaling remains unresolved, and likely depends on spatial compartmentalization and receptor context within the pulmonary vasculature.

While BMP signaling is often considered vasculoprotective, increasing evidence suggests that both insufficient and mislocalized BMP activity can contribute to vascular pathology depending on cellular context and downstream transcriptional integration. This supports a model in which PAH pathogenesis may arise not only from reduced BMP signaling but also from loss of spatial and regulatory control over BMP signaling dynamics.

Altogether, current evidence supports a limited but biologically plausible association between *GGCX* variants and PAH. However, the absence of large-scale replication studies, limited in vivo functional validation, and incomplete understanding of extracellular BMP regulation constrain causal interpretation. Within a ClinGen-aligned framework, *GGCX* is best classified as a candidate modifier gene influencing extracellular regulation of BMP signaling, whose role in pulmonary vascular disease remains to be definitively established.

### 3.7. Other Signaling Receptors

Signaling receptors are proteins located on the surface of pulmonary endothelial or smooth muscle cells that respond to external stimuli and regulate cell proliferation, growth, and differentiation. Mutations of the genes encoding for these receptors can lead to their overactivation or inhibition, shifting the balance from a stable, quiescent vessel wall to one that is progressively constricted and remodeled as in PAH.

#### 3.7.1. *NOTCH3*

*NOTCH3* is a member of the Notch receptor family that encodes a single-pass transmembrane receptor predominantly expressed in vascular smooth muscle cells, where it plays a central role in vascular development, arterial identity maintenance, and regulation of smooth muscle cell survival (Table 2). In the pulmonary circulation, *NOTCH3* is particularly relevant to pulmonary artery smooth muscle cell (PASMC) phenotype stability and proliferative capacity [78].

Following ligand binding by Jagged or Delta-like family ligands on adjacent cells, *NOTCH3* undergoes sequential proteolytic cleavage mediated by ADAM-family proteases and γ-secretase, resulting in release of the Notch3 intracellular domain (N3ICD). The N3ICD translocates to the nucleus, where it forms a transcriptional activation complex that regulates downstream targets, including the Hes and Hey (HRT) gene families. These transcriptional programs promote PASMC survival, maintain a synthetic/proliferative phenotype, and inhibit apoptosis, thereby influencing vascular remodeling capacity.

Experimental and observational studies suggest that *NOTCH3* signaling is upregulated in pulmonary arterial hypertension (PAH). Increased *NOTCH3* expression has been reported in remodeled pulmonary arteries from PAH patients, with levels correlating with disease severity, supporting an association between enhanced *NOTCH3* activity and adverse vascular remodeling [79]. Hypoxic exposure, an established trigger of pulmonary hypertension, has been shown to increase *NOTCH3* expression in experimental models, linking environmental stress to activation of this signaling axis [80]. Collectively, these findings position *NOTCH3* as a context-sensitive signaling pathway that may integrate hypoxia-driven stress responses with PASMC proliferative signaling.

Rare *NOTCH3* variants, including missense substitutions such as c.2519G>A (p.G840E) and c.2698A>C (p.T900P), have been identified in PAH cohorts [81]. Functional analyses suggest that these variants may alter *NOTCH3* signaling fidelity, including downstream transcriptional output through HES/HEY pathways, potentially contributing to dysregulated PASMC proliferation and survival. However, the available genetic evidence remains limited to small cohorts, and replication across independent PAH populations is lacking. Robust standardized functional validation of individual *NOTCH3* variants in pulmonary vascular models remains incomplete.

From a mechanistic perspective, *NOTCH3* differs from canonical PAH genes such as *BMPR2* or *SOX17* in that it primarily regulates cell fate and phenotype stability in smooth muscle cells, rather than endothelial signaling or ligand–receptor BMP pathway integrity. It therefore occupies a distinct but potentially convergent position within the broader PAH network, acting at the level of PASMC proliferation, survival, and vascular remodeling responsiveness.

A key unresolved question is whether *NOTCH3* activation represents a primary pathogenic driver, a downstream response to vascular injury (e.g., hypoxia or metabolic stress), or a secondary amplification pathway that reinforces established remodeling signals. Current evidence supports all three possibilities in different experimental contexts but does not yet allow clear causal stratification in human disease. This uncertainty is reinforced by the variability in expression patterns and the absence of large-scale genotype–phenotype correlation studies.

Within a ClinGen-consistent interpretative framework, *NOTCH3* is regarded as a candidate or emerging PAH-associated gene with supportive functional evidence but limited definitive genetic validation. Its role in pulmonary vascular disease likely reflects a modulatory pathway that amplifies PASMC proliferation and survival in response to upstream pathogenic stimuli rather than a singular initiating event. Further large-cohort genetic studies and in vivo functional modeling will be required to clarify whether *NOTCH3* contributes primarily as a disease driver, modifier, or convergence node within the broader PAH pathogenic network.

#### 3.7.2. *KDR*

*KDR* encodes vascular endothelial growth factor receptor 2 (*VEGFR2*), a receptor tyrosine kinase that mediates VEGF-driven endothelial signaling and is central to angiogenesis and vascular maintenance (Table 2). Through activation by VEGF-A, *VEGFR2* regulates endothelial proliferation, migration, survival, and permeability, placing it at the core of endothelial adaptive responses to hemodynamic stress [82].

Rare *KDR* variants have been identified in pulmonary arterial hypertension (PAH), with reported frequencies of approximately 1–2% depending on cohort composition and sequencing strategy [83]. Both missense and truncating variants have been described. Clinically, truncating variants have been associated with reduced diffusing capacity for carbon monoxide (DLCO) and later disease onset, suggesting a phenotype that reflects impaired endothelial maintenance rather than developmental vascular malformation.

Functional studies indicate that reduced *KDR* activity compromises VEGF-dependent endothelial survival signaling, leading to endothelial dysfunction and impaired repair responses under stress conditions [84]. Rather than driving proliferation directly, this loss appears to weaken endothelial resilience, making the pulmonary microvasculature less able to recover from hypoxic or inflammatory injury.

This places *VEGFR2* in a distinct but conceptually overlapping category with genes such as *SOX17* and *FOXF1*, where the primary defect is not constitutive pathway activation but reduced endothelial stability. A recurring theme across these genes is that endothelial vulnerability, rather than intrinsic hyperproliferation, lowers the threshold for vascular remodeling once secondary insults are present.

At the same time, VEGF signaling introduces a mechanistic paradox in pulmonary vascular disease. While excessive VEGF activity can promote aberrant angiogenesis in some vascular beds, insufficient VEGFR2 signaling in the lung appears to impair endothelial survival and regenerative capacity. The consequence is not simply reduced angiogenesis but a failure to maintain a stable endothelial layer under chronic stress, which may secondarily permit remodeling processes driven by other pathways such as BMP/TGF-β dysregulation.

Genetically, *KDR*-associated PAH remains incompletely defined. Penetrance is variable, reported cohorts are small, and functional validation of specific variants is still limited. These uncertainties make it difficult to determine whether *KDR* haploinsufficiency is sufficient to initiate disease or whether it acts primarily as a modifying factor within a broader multi-hit framework involving hypoxia, inflammation, or metabolic stress.

*KDR* is regarded as a limited-to-moderate confidence PAH-associated gene [15], with supportive but not definitive genetic and functional data. Its main contribution to the disease landscape appears to lie in weakening endothelial adaptive capacity rather than acting as a primary driver of proliferative remodeling.

### 3.8. Membrane/Structural Proteins

Beyond dysfunctional growth factor signaling and metabolic reprogramming, PAH also involves irregularities in structural proteins that maintain the physical architecture of vascular cells. These proteins regulate cell-to-cell interaction, barrier integrity, mechanotransduction, and vesicular trafficking, processes that are essential in maintaining vascular homeostasis. The two structural proteins implicated in PAH are caveolin-1 and pleckstrin homology domain-containing protein family H member 2.

#### 3.8.1. *CAV1*

*CAV1* encodes caveolin-1, the principal structural protein of caveolae—plasma membrane invaginations involved in endocytosis, mechanosensing, and spatial organization of signaling complexes in endothelial and smooth muscle cells (Figure 2) [85,86]. By organizing receptor localization and trafficking, caveolin-1 regulates multiple signaling pathways that control endothelial survival, proliferation, and vascular tone, placing it at a central regulatory node in pulmonary vascular homeostasis.

Pathogenic variants in *CAV1* were first implicated in pulmonary arterial hypertension (PAH) through the identification of loss-of-function mutations in familial and idiopathic cases [87]. These findings established *CAV1* as a rare but biologically plausible PAH-associated gene. Subsequent studies in patient-derived samples and experimental systems confirmed reduced caveolin-1 expression in pulmonary vascular tissue, supporting a disease-associated deficiency state rather than simple receptor-level dysregulation [87].

Caveolin-1 deficiency disrupts membrane microdomain organization and alters receptor compartmentalization, leading to dysregulated signaling through multiple pathways, including BMP and TGF-β signaling cascades. In pulmonary vascular cells, this imbalance favors enhanced TGF-β-driven proliferative signaling and reduced BMP-mediated quiescence, a pattern consistent with vascular remodeling phenotypes observed in established PAH genes such as *BMPR2* and *ACVRL1* [87]. However, unlike receptor or ligand mutations, *CAV1* does not directly impair ligand binding or SMAD phosphorylation; instead, it modifies the spatial and temporal dynamics of signaling, amplifying downstream pathway dysregulation in a context-dependent manner.

In endothelial cells, *CAV1* loss impairs caveolae-dependent mechanotransduction and nitric oxide signaling, contributing to endothelial dysfunction and barrier instability. In smooth muscle cells, altered signaling compartmentalization promotes a pro-proliferative, apoptosis-resistant phenotype. These dual effects provide a mechanistic bridge between endothelial injury and smooth muscle remodeling, although the relative contribution of each compartment remains incompletely defined in human PAH tissue.

Key uncertainties nonetheless remain. Although *CAV1* is widely expressed across multiple tissues, pathogenic variants predominantly manifest as pulmonary vascular disease, suggesting tissue-specific vulnerability. One proposed explanation is the unique dependence of the pulmonary circulation on finely tuned mechanosensing and growth factor signaling under conditions of chronic shear stress. However, direct human in vivo evidence supporting pulmonary selectivity is still limited, and most mechanistic data derive from murine models and cultured endothelial systems [85,87].

Within current evidence frameworks, *CAV1* is classified as a definitive PAH gene by the Clinical Genome Resource Pulmonary Hypertension Gene Curation Expert Panel [15]. Unlike canonical BMP/TGF-β ligands or receptors, caveolin-1 does not directly regulate signal initiation but instead controls the spatial organization and trafficking of multiple signaling complexes at the plasma membrane. This positions *CAV1* as a regulator of signaling architecture, where loss of membrane compartmentalization amplifies dysregulated pathways rather than initiating them de novo. In contrast to genes such as *KDR* or *AQP1*, which remain at moderate or emerging levels of evidence, *CAV1* illustrates how disruption of membrane-level signal integration can represent a fully established pathogenic mechanism in pulmonary arterial hypertension.

#### 3.8.2. *PLEKHH2*

*PLEKHH2* is located on chromosome 2p21 and encodes a cytoskeletal adaptor protein involved in cell–matrix adhesion and structural stabilization of specialized barrier-forming cells (Table 2) [88]. In renal tissue, it has been characterized in podocytes, where it contributes to maintenance of foot process architecture and anchoring to the glomerular basement membrane, a structure essential for filtration barrier integrity. This functional role in highly specialized barrier cells suggests that *PLEKHH2* participates in maintaining mechanical stability in vascular and epithelial interfaces.

Beyond the kidney, *PLEKHH2* is expressed in pulmonary vascular endothelial cells, where emerging evidence suggests a role in maintaining endothelial structural integrity and homeostatic signaling [89]. This shared expression across podocytes and pulmonary endothelium supports the broader concept that barrier-forming cells rely on conserved cytoskeletal–matrix coupling systems to withstand mechanical stress and maintain selective permeability.

Rare missense variants in *PLEKHH2* have recently been identified in pulmonary arterial hypertension (PAH) cohorts and have been associated with increased endothelial proliferation and reduced apoptosis in experimental endothelial models [89]. These observations suggest that *PLEKHH2* disruption may destabilize endothelial anchorage to the extracellular matrix, thereby altering mechanotransduction pathways that normally regulate survival and controlled cell cycling under shear stress conditions.

Unlike canonical PAH genes such as *BMPR2* or *ACVRL1*, *PLEKHH2* does not directly participate in ligand–receptor signaling or intracellular SMAD-mediated transcriptional regulation. Instead, its putative role is structural, influencing how endothelial cells sense and respond to mechanical cues from the extracellular matrix. In this sense, it aligns with a growing subset of PAH-associated genes, including *ATP13A3*, *AQP1*, *CAV1*, and *SOX17*, where the primary defect is not constitutive pathway activation but reduced endothelial resilience to environmental and mechanical stressors.

This structural vulnerability model helps reconcile the observed phenotype: impaired cytoskeletal anchoring may not directly induce proliferation but may lower the threshold for maladaptive responses when endothelial cells are exposed to secondary insults such as hypoxia, inflammation, or dysregulated BMP/TGF-β signaling. However, current evidence does not establish *PLEKHH2* as an initiating driver of disease; instead, it is more consistent with a modifier role that amplifies susceptibility to vascular remodeling.

Evidence supporting *PLEKHH2* involvement in PAH remains preliminary. The number of reported pathogenic variants is still small, functional studies are largely limited to in vitro endothelial systems, and in vivo validation in pulmonary vascular models is currently lacking. As a result, genotype–phenotype correlations remain uncertain, and disease penetrance cannot yet be reliably estimated.

### 3.9. Vasoactive Regulation

PAH involves a dysregulated balance between vasodilators (downregulated) and vasoconstrictors (upregulated), contributing to the elevated pulmonary artery pressure and resistance. The *KLK1* gene, which plays a crucial role in regulating vascular tone through the kallikrein–kinin system, has been implicated in the distortion of this vasodilator-vasoconstrictor balance.

#### *KLK1* 

*KLK1* encodes tissue kallikrein, a secreted serine protease of the kallikrein family that is widely expressed in multiple organs, including the lung, kidney, heart, and pancreas [90]. Its primary physiological role is the generation of kinins, particularly bradykinin, through cleavage of kininogen substrates. Bradykinin signaling promotes vasodilation via nitric oxide and prostacyclin release, and contributes to endothelial repair, angiogenesis, and modulation of vascular tone, positioning *KLK1* within a protective endothelial signaling axis in normal pulmonary vascular physiology.

Rare *KLK1* variants have been reported in pulmonary arterial hypertension (PAH) cohorts, with an estimated frequency of approximately 0.5% in selected genetic studies [75]. However, the available evidence remains limited in scale and replication. Clinically, carriers have been described as presenting at older ages with comparatively milder phenotypes than *BMPR2*-associated disease, suggesting a weaker or modulatory contribution to disease susceptibility rather than a high-penetrance pathogenic effect.

*KLK1* deficiency is thought to impair the local kinin–bradykinin axis, reducing nitric oxide-mediated vasodilation and endothelial repair signaling. Rather than directly promoting proliferative remodeling, this mechanism may weaken adaptive responses to vascular injury, particularly in distal pulmonary arterioles, where regenerative capacity is critical. In this sense, *KLK1* dysfunction is more consistent with impaired vascular maintenance and repair than with primary activation of proliferative signaling pathways.

This distinguishes *KLK1* from canonical PAH genes involved in BMP/TGF-β signaling, ion channel regulation, or metabolic stress responses. Instead, its functional profile aligns more closely with endothelial competence and angiogenic support genes such as *KDR* and *FOXF1*, where disease risk arises from reduced ability to maintain or restore vascular integrity under stress conditions. This supports a broader view of PAH pathobiology in which impaired vascular regeneration contributes alongside excessive vasoconstrictive or proliferative signaling.

An additional modifying layer may involve hormonal regulation. Experimental studies have shown that estrogen can upregulate *KLK1* expression [91], raising the possibility that sex-dependent modulation of the kallikrein–kinin system contributes to variability in disease expression. This interaction has been proposed as one potential contributor to sex bias in PAH cohorts, although direct mechanistic evidence in *KLK1*-associated disease remains limited and should be interpreted cautiously.

Despite these associations, current evidence is constrained by small patient numbers, limited replication across independent cohorts, and a lack of robust functional validation in human pulmonary vascular systems. It therefore remains unresolved whether *KLK1* variants can independently initiate disease or whether they primarily act as low-penetrance modifiers that reduce endothelial repair capacity in the presence of additional genetic or environmental stressors.

Within a ClinGen-consistent framework, *KLK1* is best regarded as a limited-evidence, candidate modifier gene [15], with a mechanistic role centered on endothelial repair signaling rather than primary pathway disruption.

### 3.10. Extracellular/Matrix Related

Recent genetic studies have identified *CBLN2* and *CD248* as active drivers of PAH and not just passive consequences of vascular injury. Extracellular matrix remodeling contributes to PAH by influencing stiffness, signaling, mechanotransduction, and recruitment of inflammatory cells in the pulmonary vasculature. These findings underscore the role of adventitial fibroblasts, in addition to endothelial and pulmonary artery smooth muscle cells, in PAH pathogenesis.

#### 3.10.1. *CBLN2*

*CBLN2* (Cerebellin 2) is a member of the cerebellin glycoprotein family, which is predominantly expressed in the central nervous system but has also been detected in pulmonary tissue, including lungs from patients with pulmonary arterial hypertension (PAH) (Table 2). This atypical expression pattern in a non-neuronal vascular context suggests a stress-associated reactivation or dysregulation of developmental signaling programs in diseased pulmonary endothelium.

A common regulatory variant (rs2217560 G allele) within the *CBLN2* locus (18q22.3) has been associated with PAH, with a reported odds ratio of approximately 2.16 in case–control analyses [92]. In addition to genetic association, increased *CBLN2* mRNA expression has been observed in PAH lung tissue, with preferential enrichment in endothelial cells compared with pulmonary artery smooth muscle cells. This cell-type specificity suggests that *CBLN2* may act primarily within the endothelial compartment during disease development.

In pulmonary vascular cells, *CBLN2* has been implicated in the activation of inflammatory and hypoxia-responsive signaling pathways, including NF-κB, *HIF-1α*, and TWIST1, which converge on endothelial-to-mesenchymal transition (EndMT) programs [93]. EndMT is increasingly recognized as a key contributor to pulmonary vascular remodeling, linking inflammatory stress and hypoxic signaling to endothelial plasticity, loss of barrier integrity, and acquisition of mesenchymal-like features. Through this mechanism, *CBLN2* is positioned within a broader network of PAH-associated genes that influence endothelial phenotype stability, including *SOX17*, *CAV1*, *KDR*, and *NOTCH3*, although it operates through distinct upstream regulatory inputs.

Unlike canonical PAH genes that directly regulate BMP/TGF-β signaling, ion channel activity, or mitochondrial metabolism, *CBLN2* appears to function as an inflammation- and hypoxia-responsive modulator of endothelial fate. Its activity is therefore more consistent with a disease-amplifying role, in which pre-existing vascular stressors promote maladaptive transcriptional reprogramming rather than *CBLN2* acting as a primary initiating lesion.

However, the current evidence base remains preliminary. The initial genetic association study was limited by moderate cohort size, and subsequent mechanistic insights are largely derived from in vitro signaling and endothelial transition models. Robust in vivo functional validation in pulmonary vascular systems, as well as replication in independent large-scale genetic cohorts, is still lacking. Consequently, it remains uncertain whether *CBLN2* represents a true disease gene, a context-dependent modifier of endothelial injury responses, or a downstream marker of inflammatory endothelial activation.

#### 3.10.2. *CD248*

*CD248* (also known as endosialin or tumor endothelial marker 1) encodes a type I transmembrane glycoprotein belonging to the C-type lectin-like receptor family, with established roles in mesenchymal–vascular interactions during development (Table 2). During embryogenesis, *CD248* is widely expressed in angiogenic vasculature, but its expression becomes largely silenced in most adult tissues, persisting mainly in specialized stromal and perivascular compartments such as fibroblasts and pericyte-rich vascular niches [94,95] dely expressed in angiogenic vasculature, but its expression becomes largely silenced in most adult tissues, persisting mainly in specialized stromal and perivascular compartments such as fibroblasts and pericyte-rich vascular niches.

Emerging genetic and experimental evidence implicates *CD248* in pulmonary arterial hypertension (PAH), although the data remain limited. Rare variants and altered expression patterns have been reported in PAH cohorts, alongside observations of reduced circulating *CD248* levels and decreased tissue expression in pulmonary vascular lesions [96]. These findings suggest that *CD248* deficiency may be associated with impaired vascular integrity, although causality has not been firmly established.

*CD248* is thought to regulate pericyte behavior, including proliferation, migration, and interaction with endothelial cells. Disruption of this signaling axis may impair pericyte–endothelial communication, weakening microvascular stability and contributing to aberrant vascular remodeling [96]. In the pulmonary circulation, where pericyte–endothelial coupling is essential for maintaining vessel caliber and resistance, such disruption could lower the threshold for endothelial dysfunction and progressive occlusive remodeling.

Unlike canonical PAH genes involved in BMP/TGF-β signaling, ion channel regulation, or metabolic stress responses, *CD248* does not directly participate in intracellular signaling cascades. Instead, it operates at the level of vascular structural organization and cell–cell communication, particularly within the perivascular compartment. This places *CD248* in a broader category of vascular support genes that influence disease susceptibility indirectly by modulating the stability of the endothelial–pericyte unit, conceptually aligning with genes involved in endothelial resilience and vascular niche maintenance.

However, the current evidence base remains preliminary. The number of reported pathogenic variants is small, cohort sizes are limited, and functional studies are largely observational or in vitro in nature. In addition, the developmental regulation of *CD248* raises unresolved questions regarding its contribution to adult-onset PAH, particularly whether disease-associated alterations represent pathological reactivation of developmental programs or primary adult-onset dysregulation in pulmonary vascular cells. This distinction remains to be established through systematic genetic and functional studies in adult pulmonary vascular models.

### 3.11. Epigenetic/Nuclear Regulation

Dysregulated epigenetic and nuclear mechanisms suppress vasoprotective gene expression, triggering pulmonary vascular remodeling, a hallmark feature of PAH. *TET2* (epigenetic dysregulation) and *TOPBP1* (DNA damage) mutations have been identified to contribute to PAH.

#### 3.11.1. *TET2*

The *TET2* gene, located on chromosome 4q24, encodes a Fe(II)/α-ketoglutarate-dependent dioxygenase that catalyzes the conversion of 5-methylcytosine to 5-hydroxymethylcytosine, thereby enabling active DNA demethylation and dynamic epigenetic regulation (Table 2). Through this function, *TET2* contributes to maintaining transcriptional programs that preserve endothelial quiescence and immune balance. Epigenetic homeostasis is governed by the opposing activities of DNA methyltransferases and TET enzymes; disruption of this balance may shift vascular cells toward persistent transcriptional repression of protective pathways [97].

Rare *TET2* variants, predominantly germline in reported PAH cohorts, account for approximately 0.39% of cases [98]. Most reported pathogenic variants cluster within the *TET2* expression has been demonstrated in peripheral blood mononuclear cells from affected individuals, alongside increased expression of pro-inflammatory mediators, particularly interleukin-1β (IL-1β).

Mechanistic evidence from experimental models suggests that *TET2* deficiency may contribute to pulmonary vascular disease through an inflammation-linked epigenetic mechanism. Loss of *TET2* activity is associated with increased DNA methylation and sustained activation of inflammatory signaling, while pharmacological inhibition of IL-1β has been shown to attenuate pulmonary hypertension phenotypes in animal models. IL-1β promotes pulmonary artery smooth muscle cell proliferation and migration [99], providing a biologically plausible link between epigenetic dysregulation and vascular remodeling. These findings place *TET2* within an inflammatory–epigenetic axis that overlaps conceptually with genes such as *EIF2AK4* and *CBLN2*, which also connect stress or inflammatory signaling to vascular dysfunction.

In contrast to canonical PAH genes involved in BMP/TGF-β signaling, ion channel function, or mitochondrial metabolism, *TET2* acts as a broad epigenetic regulator capable of influencing multiple downstream pathways simultaneously. This positions *TET2* more as a modifier of vascular transcriptional responsiveness rather than a pathway-specific signaling defect. Such a role is consistent with its association with later-onset disease and systemic inflammatory features, suggesting that epigenetic drift may lower the threshold for vascular remodeling rather than directly initiating it.

However, the current evidence base remains limited by small patient numbers and a reliance on peripheral blood and animal model data, with few studies directly examining pulmonary vascular tissue. It therefore remains unclear whether *TET2* dysfunction primarily originates within the pulmonary endothelium or reflects systemic immune-driven epigenetic reprogramming that secondarily impacts the lung circulation. Future integrative studies combining pulmonary epigenomics, single-cell profiling, and longitudinal clinical data will be essential to define whether *TET2* acts as a primary disease contributor or an amplifier within established inflammatory and remodeling pathways in PAH.

#### 3.11.2. *TOPBP1*

Topoisomerase IIβ-binding protein 1 (*TOPBP1*), located on chromosome 3, encodes a multifunctional scaffold protein that regulates DNA replication initiation, ATR-dependent checkpoint activation, and DNA damage repair (Table 2). Through its BRCA1 C-terminal (BRCT) domains, *TOPBP1* mediates phospho-protein interactions required for assembly of DNA damage response complexes, thereby preserving genomic stability during replicative stress. Biochemical studies demonstrated that *TOPBP1* directly activates ATR kinase via its ATR-activation domain, establishing its central role in DNA damage checkpoint signaling [100], while structural and functional analyses have shown that BRCT domains enable selective binding to phosphorylated substrates at sites of DNA damage [101]. This function is particularly relevant in vascular endothelial cells, which are continuously exposed to shear stress and oxidative injury that can induce DNA damage.

Whole-exome sequencing identified several single-nucleotide variants in *TOPBP1* (rs55633281, rs17301766, rs10935070) in idiopathic PAH patients lacking *BMPR2* mutations [102]. However, these observations were derived from a relatively small cohort and have not been independently replicated. Consistent with this limited evidence base, *TOPBP1* is not currently classified as a PAH-associated gene by the Clinical Genome Resource [15] and is therefore best considered a candidate susceptibility gene rather than a confirmed causal factor.

At the cellular level, reduced *TOPBP1* expression has been reported in pulmonary microvascular endothelial cells from PAH patients, accompanied by increased DNA damage and attenuated apoptotic responses. Functional knockdown experiments further demonstrated accumulation of DNA damage alongside persistence of apoptosis-resistant cells, suggesting impaired genomic quality control. Rather than directly promoting proliferation, these findings support a model in which defective DNA damage surveillance permits survival of dysfunctional endothelial cells, thereby compromising vascular integrity and predisposing to maladaptive remodeling.

In contrast to established PAH genes such as *BMPR2*, *SOX17*, and *KDR*, which have well-defined roles in endothelial signaling, angiogenesis, and vascular homeostasis, the evidence supporting *TOPBP1* remains limited and largely hypothesis-generating. Its proposed role instead aligns more closely with genes such as *FOXF1* and *TET2*, which influence endothelial resilience through regulation of genomic stability and epigenetic integrity rather than direct modulation of proliferative signaling pathways.

A key conceptual challenge is reconciling *TOPBP1*-associated genomic instability with the hyperproliferative vascular lesions characteristic of PAH. One plausible explanation is that accumulation of DNA-damaged but apoptosis-resistant endothelial cells promotes maladaptive repair responses, inflammation, or clonal expansion. Alternatively, localized endothelial injury may trigger compensatory proliferation in neighboring vascular cells. These mechanisms remain speculative and have not been validated in pulmonary vascular models.

In summary, the current evidence base is constrained by small sample sizes, lack of independent replication, and reliance on in vitro endothelial systems. In vivo studies examining endothelial-specific *TOPBP1* dysfunction in pulmonary vascular contexts are lacking, and genotype–phenotype relationships remain undefined. Accordingly, *TOPBP1* is best interpreted as a biologically plausible but genetically unvalidated contributor, highlighting a potential genomic instability axis in PAH that requires further investigation rather than representing an established pathogenic driver.

### 3.12. Single-Nucleotide Polymorphisms (SNPs)

SNPs, being more common than rare variants, often act as modifiers of PAH susceptibility, severity, or response to treatment. SNPs in the *TGF-β1* gene (509 and codon 10) of familial PAH patients with known *BMPR2* mutations are associated with earlier onsets and increased penetrance of the disease [7] (Table 1). This is attributed to the resultant imbalance of the TGF-β and BMP signaling pathways that trigger aberrant vascular cell proliferation and apoptosis resistance.

**Table 1:** Single-Nucleotide Polymorphisms associated with PAH.

Gene	SNP Location	Proposed Mechanism Leading to PAH	References
*TGF-β1*	Chromosome 19 (509 C>T, Codon 10 T>C)	Imbalanced TGF-β/BMP signaling with enhanced TGF-β-mediated SMAD2/3 activation, promoting endothelial dysfunction, PASMC proliferation, extracellular matrix remodeling, and vascular fibrosis.	[7]
*SOX17*	Chromosome 8 (rs13266183, rs10103692)	Impaired endothelial SOX17 signaling leading to dysregulated Wnt/β-catenin signaling, endothelial dysfunction, defective angiogenesis, and pulmonary vascular remodeling.	[103]
*GNG2*	Chromosome 14 (rs11157866)	Aberrant G-protein signaling with enhanced endothelin-1-mediated vasoconstrictive and proliferative pathways contributing to pulmonary vascular remodeling.	[104]
*KCNA5*	Chromosome 12 (rs10744676)	Loss of Kv1.5 channel function causing membrane depolarization, reduced K^+^ efflux, increased intracellular Ca^2+^, PASMC proliferation, and apoptosis resistance.	[105]
*HIF1A*	Chromosome 14 (rs12434438)	Enhanced HIF-1α activity induces pyruvate dehydrogenase kinase-1 (PDK1), suppressing mitochondrial oxidative phosphorylation and promoting aerobic glycolysis, PASMC hyperproliferation, and apoptosis resistance.	[106,107]
*SIRT3*	Chromosome 11	Reduced mitochondrial deacetylase activity causing hyperacetylation of mitochondrial enzymes, impaired oxidative phosphorylation, increased oxidative stress, and metabolic reprogramming toward aerobic glycolysis in PASMCs.	[108]
*UCP2*	Chromosome 11	Mitochondrial dysfunction and excess reactive oxygen species (ROS) generation stabilize HIF-1α signaling, promoting PASMC proliferation, apoptosis resistance, and pulmonary vascular remodeling.	[108]

### 3.13. Non-Coding RNAs

Long non-coding RNAs (lncRNAs), which are RNA molecules >200 nucleotides in length and lack protein-coding ability, have also been implicated in PAH pathogenesis. lncRNAs such as tyrosine kinase inducing receptor lncRNA (TYKRIL), H19, lncRNA cancer susceptibility candidate 2 (CASC2), lncTNA taurine-upregulated gene 1 (TUG1), and paxip1 antisense RNA1 (PAXIP1-AS1) contribute to PAH by inducing uncontrolled proliferation, survival, and migration of pulmonary artery endothelial and smooth muscle cells [109]. LncRNAs are also associated with oxidative stress and metabolism dysregulation, established drivers in PAH pathogenesis.

The genes involved in the pathogenesis of PAH are summarized in Table 2.

**Table 2:** Genes involved in the pathogenesis of PAH.

Grouping According to Function	Gene Symbol	Location	Product Name	Function	References
Receptors of the TGF-β family	*BMPR2*	2q33 (13 exons)	BMPRII	The BMPR2 receptor, phosphorylates type I receptors upon ligand binding, which in turn activate the SMAD1/5/8 pathway (BMP signaling). BMP signaling regulates smooth muscle and endothelial cell proliferation and migration. It also modulates inflammation through decreased expression of interleukin 6, tumor necrosis factor alpha, and monocyte chemoattractant protein 1.	[10,110]
	*ACVRL1*	12q13.13 (11 exons)	*ALK1*	*ALK1*, encoded by *ACVRL1*, functions as a modulator of BMP pathway signaling.	[111]
	*ENG*	9q34.11 (15 exons)	Endoglin	Encoded by ENG, the integral membrane protein Endoglin plays an important role in angiogenesis.	[33]
	*BMPR1B*	4q22.3 (21 exons)	*BMPR1B* Recombinant	Essential in BMP signaling, this receptor activates R-SMADs (1/5/8).	[36]
Ligands of the BMP signaling pathway	*GDF2*	10q11.22 (2 exons)	BMP-9	BMP-9, encoded by *GDF2*, acts as a ligand for the BMPRII receptor, with receptor binding stimulating BMP signaling.	[41]
	*BMP10*	2p13.3 (2 exons)	BMP-10	BMP10, primarily produced in the right atrium, is essential for cardiac development and angiogenesis.	[43]
Transcription factors	*TBX4*	17q23.2 (11 exons)	*TBX4*	TBX4, a T-box transcription factor encoded by *TBX4*, directly regulates the promoter of fibroblast growth factor 10 (*FGF10*), which is pivotal in the development of the lungs and pulmonary vascular beds.	[45,46]
	*SOX17*	8q11.23 (2 exons)	*SOX17*	*SOX17* plays an important role in embryonic development, particularly in cardiogenesis and postnatal vascular remodeling.	[112]
	*KLF4*	9q31 (5 exons)	KLF Transcription Factor 4	KLF4, a modulator of angiogenesis, preserves adherens junction integrity to prevent vascular leakage and also inhibits epithelial-to-mesenchymal transition.	[51]
	*FOXF1*	16q24.1 (2 exons)	Foxhead box protein F1	*FOXF1* encodes forkhead box protein F1, which is involved in embryonic angiogenesis and DNA repair within the pulmonary vasculature.	[53,54]
	*SMAD1*	4q31.21 (12 exons)	*SMAD1*	SMAD1, upon phosphorylation by type I receptors, complexes with SMAD4 and translocates to the nucleus.	[44]
	*SMAD4*	18q21.2 (12 exons)	*SMAD4*	SMAD4 functions as the common co-SMAD that binds phosphorylated SMAD1/5/8 and SMAD2/3 complexes before their translocation to the nucleus to regulate target gene transcription.	[44]
	*SMAD8*	13q13.3 (10 exons)	SMAD8	SMAD8 acts as a downstream modulator of BMP signaling; following activation by BMP type I receptors, it complexes with SMAD4 and translocates to the nucleus to regulate gene expression.	[56]
Membrane Transporters	*ATP13A3*	3q29 (36 exons)	ATPase13A3	*ATP13A3* is predominantly expressed in recycling endosomes and plays an essential role in polyamine metabolism and uptake.	[58]
	*AQP1*	7p14.3 (4 exons)	Aquaporin-1	*AQP1* encodes aquaporin-1, a membrane protein that regulates water transport across the cell membrane.	[62]
Potassium ion transporters	*KCNA5*	12p13 (1 exon)	Potassium voltage-gated channel subfamily A member 5	*KCNA5* encodes the Kv1.5 channel α-subunit, which plays an important role in maintaining pulmonary vascular tone.	[64]
	*KCNK3*	2p23.3 (3 exons)	TASK-1	*KCNK3* encodes the TASK1 potassium channel protein, which regulates the resting membrane potential in various tissues.	[66]
	*ABCC8*	11p15.1 (38 exons)	SUR1	*ABCC8* encodes SUR1, a regulatory subunit of the ATP-sensitive potassium channel.	[67]
Metabolic genes	*EIF2AK4*	15q15.1 (39 exons)	GCN2	*EIF2AK4* encodes GCN2, a kinase that phosphorylates EIF2α to regulate protein synthesis and function as a sensor of cellular stress.	[70]
	*NFU1*	2q36.1 (8 exons)	*NFU1* iron–sulfur cluster scaffold	*NFU1* encodes a protein essential for mitochondrial Fe–S cluster assembly, with these clusters serving as critical cofactors in the electron transport chain.	[73]
	*GGCX*	2p11.2 (15 exons)	*GGCX*	*GGCX* encodes gamma-glutamyl carboxylase, an enzyme crucial for regulating coagulation, cell proliferation, and inflammation.	[75]
Other Signaling receptors	*NOTCH3*	19p13.12 (33 exons)	Notch 3 protein	*NOTCH3* encodes a transmembrane protein that is essential for blood vessel maintenance by regulating the survival and function of vascular smooth muscle cells.	[78]
	*KDR*	4q12 (30 exons)	*VEGFR2*	VEGFR2 is encoded by *KDR* and is essential for angiogenesis, endothelial cell proliferation, survival, and migration.	[84]
Membrane/structural proteins	*CAV1*	7q31.2 (4 exons)	Caveolin-1	Caveolin-1, encoded by *CAV1*, is a structural component of caveolae that are crucial for endocytosis, mechanosensing, and the spatial organization of signaling complexes.	[87]
	*PLEKHH2*	2p21 (30 exons)	*PLEKHH2*	*PLEKHH2* encodes a protein that links podocyte foot processes to the glomerular basement membrane, supporting renal filtration, and is also implicated in the regulation of endothelial cell proliferation and apoptosis.	[88,89]
Vasoactive regulation	*KLK1*	19q13.33 (6 exons)	Kallikrein 1	*KLK1* encodes tissue kallikrein, which regulates blood pressure, inflammation, cell proliferation, and vascular tone.	[90]
Extracellular/matrix-related	*CBLN2*	18q22.3 (3 exons)	Cerebellin 2 Precursor	*CBLN2* encodes cerebellin-2, a protein responsible for maintaining synaptic integrity and function.	[92]
	*CD248*	11q13.2 (1 exon)	Endosialin/Tumor Endothelial Marker 1	CD248 encodes the CD248 protein essential for embryonic angiogenesis and for maintaining vascular tone and pulmonary homeostasis.	[94,96]
Epigenetic/nuclear regulation	*TET2*	4q24 (11 exons)	*TET2*	*TET2* encodes the TET2 protein, an enzyme that regulates DNA methylation and plays a key role in epigenetic programming.	[97,98]
	*TOPBP1*	3q22.1 (28 exons)	*TOPBP1*	*TOPBP1* encodes a DNA topoisomerase II-binding protein that plays a crucial role in angiogenesis, DNA replication, and DNA repair.	[102]

## 4. Contribution of New Technologies and Multi-Omics to Expanding the Genetic Architecture of PAH

**Figure 4:**
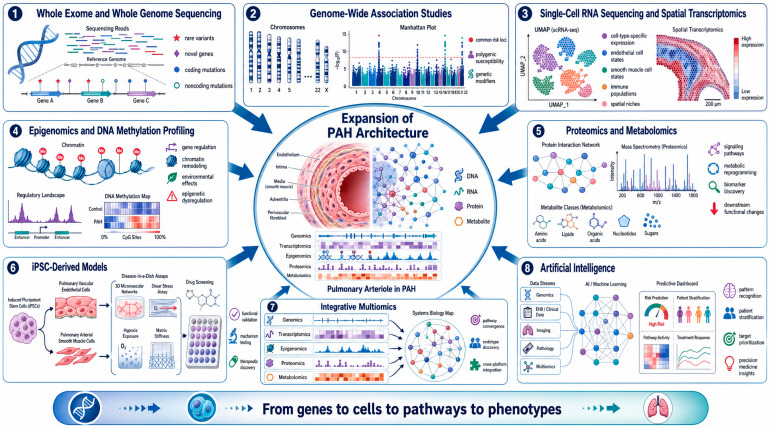
Emerging technologies expanding the genetic architecture of pulmonary arterial hypertension (PAH). Schematic overview of complementary technologies used to investigate genotype–phenotype relationships in PAH. ① Whole-exome and whole-genome sequencing identify rare coding and non-coding variants associated with disease susceptibility. ② Genome-wide association studies (GWAS) detect common risk loci and polygenic modifiers. ③ Single-cell RNA sequencing and spatial transcriptomics resolve cell-type-specific and spatially localized molecular programs within remodeled pulmonary vasculature. ④ Epigenomic profiling identifies regulatory alterations associated with gene dysregulation. ⑤ Proteomics and metabolomics characterize downstream signaling and metabolic changes. ⑥ iPSC-derived vascular models enable mechanistic investigation and therapeutic testing of PAH-associated variants. ⑦ Integrative multi-omics frameworks combine multidimensional datasets to identify convergent pathogenic pathways. ⑧ Artificial intelligence and machine learning approaches facilitate systems-level analysis, patient stratification, and precision medicine applications. This figure was created by the authors using ConceptViz, a web-based concept visualization tool. No external databases or proprietary graphical resources were used.

The genetic architecture of PAH has historically been defined through candidate-gene studies and linkage analyses in familial cohorts. Technological advances in next-generation sequencing, multi-omics platforms, and patient-derived cellular models have fundamentally expanded the known PAH gene catalog, illuminated molecular mechanisms extending well beyond the canonical BMP/TGF-β axis, and begun to explain the incomplete penetrance and phenotypic heterogeneity that characterize this disease (Figure 4). Critically, the contribution of these technologies is considered here at the methodological and systems level; the specific gene-level discoveries enabled by each platform are discussed in detail within the individual gene sections above.

### 4.1. Whole-Exome and Whole-Genome Sequencing

Whole-exome sequencing (WES) transformed PAH gene discovery by enabling unbiased interrogation of the protein-coding genome across large patient cohorts. Applied to 2572 PAH patients by [75] through the PAH Biobank, WES extended the genetic diagnostic yield from approximately 20% (attributable to BMPR2 alone) to nearly 30%, identifying nine novel risk genes simultaneously. This single study illustrated the superiority of WES over sequential candidate-gene testing for rare disease gene discovery by allowing simultaneous interrogation of thousands of coding regions without reliance on prior mechanistic assumptions. Importantly, WES revealed that the PAH gene landscape encompasses not only BMP/TGF-β signaling receptors but also polyamine transporters, water channels, vascular growth factor receptors, epigenetic regulators, and coagulation enzymes, classes of genes that would not have been included in hypothesis-driven panels [75]. However, despite its transformative contribution to rare variant discovery, WES remains intrinsically limited by incomplete exon capture, poor coverage of GC-rich regions, and inability to reliably detect structural variants, repeat expansions, or pathogenic regulatory mutations outside protein-coding regions. Consequently, a substantial proportion of heritable PAH remains genetically unresolved even in large exome cohorts, highlighting the limitations of coding-centric approaches for fully defining disease architecture.

Whole-genome sequencing (WGS) extends this reach by capturing non-coding variants, deep intronic changes creating cryptic splice sites, and structural rearrangements invisible to exome-centric approaches. WGS analyses performed within the NIHR BioResource–Rare Diseases program identified pathogenic deep-intronic *BMPR2* variants in exome-negative familial PAH cases, resolving the genetic cause in a subset of families previously classified as idiopathic [4]. Compared with WES, WGS provides more uniform genomic coverage and improved detection of structural and regulatory variation, making it particularly valuable for unresolved familial disease. Nevertheless, interpretation of non-coding variation remains a major analytical challenge because the functional consequences of most intronic and intergenic variants remain poorly annotated in pulmonary vascular tissues. In addition, the substantially higher financial, computational, and bioinformatic demands of WGS currently limit its widespread clinical implementation relative to WES. As WGS datasets mature and are integrated with epigenomic and chromatin accessibility maps, interpretation of non-coding variants at disease-relevant regulatory elements will become an increasingly important frontier for expanding the PAH genetic architecture. Together, WES and WGS are increasingly viewed as complementary rather than competing technologies, with WES providing cost-effective large-scale coding variant discovery and WGS enabling comprehensive interrogation of unresolved non-coding and structural genetic variation.

### 4.2. Genome-Wide Association Studies and Polygenic Architecture

Genome-wide association studies (GWAS) have revealed that common polymorphisms contribute to PAH susceptibility at the population level, complementing rare variant discovery by identifying disease-relevant loci that would not segregate in family-based studies. The international GWAS meta-analysis by [92,103], encompassing 2085 cases and over 9000 controls, identified genome-wide significant loci at SOX17 (chromosome 8q11.23) and HLA-DPA1/DPB1 (chromosome 6p21), implicating endothelial transcriptional dysregulation and immune-mediated mechanisms as common pathogenic themes. An earlier GWAS by identified a susceptibility locus at *CBLN2* (18q22.3). Unlike WES and WGS, which are optimized for detection of rare, high-penetrance variants in familial disease, GWAS is specifically designed to detect common low-effect polymorphisms contributing to population-level disease susceptibility. This complementary methodological framework has broadened understanding of PAH beyond monogenic inheritance by implicating regulatory and immune-related pathways that may modify disease risk across genetically diverse populations.

Crucially, the convergence of GWAS loci with rare variant genes, most notably *SOX17*, which harbors both common risk variants and rare loss-of-function mutations, demonstrates that PAH genetic risk is distributed across the allele frequency spectrum. This convergence supports a polygenic component in which common variants at disease-relevant loci modify penetrance and expressivity of rare high-impact mutations, providing a framework for understanding why carriers of identical *BMPR2* mutations can manifest strikingly different disease severity [92,103]. However, despite their statistical power for detecting susceptibility loci, GWAS findings typically confer modest individual effect sizes and frequently localize to non-coding regions, making causal gene assignment and mechanistic interpretation challenging. In contrast to rare variant sequencing, which can directly identify pathogenic mutations, GWAS often requires integration with epigenomic mapping, chromatin accessibility profiling, and transcriptomic datasets to establish biological relevance. Furthermore, because PAH is a rare disease, GWAS studies remain constrained by relatively limited cohort sizes compared with more common complex diseases, reducing power to detect additional low-frequency susceptibility loci. Consequently, the greatest insight into PAH genetic architecture has emerged not from GWAS or sequencing independently, but from their convergence, where common variant associations, rare pathogenic mutations, and downstream molecular profiling collectively reveal interconnected pathways governing endothelial dysfunction, immune activation, and vascular remodeling.

### 4.3. Single-Cell RNA Sequencing and Spatial Transcriptomics

Single-cell RNA sequencing (scRNA-seq) has addressed a fundamental limitation of bulk transcriptomics—the averaging of signals across heterogeneous cell populations—by enabling transcriptomic profiling at individual cell resolution. In PAH, scRNA-seq applied to patient lung tissue has resolved disease-associated endothelial subpopulations characterized by transcriptional signatures of endothelial-to-mesenchymal transition (EndMT), impaired quiescence, and inflammatory activation. These cell states provide a cellular framework for contextualizing where and in which cell types PAH risk genes, including *SOX17*, *FOXF1*, *KLF4*, and *TET2*, exert their primary pathogenic effects [113]. scRNA-seq has also resolved the immune cell landscape of PAH lungs, identifying macrophage and T-cell subpopulations that drive IL-1β-mediated vascular inflammation, providing cellular context for the *TET2*-driven inflammatory axis described by [97,98]. Compared with bulk RNA sequencing, which provides broad transcriptomic coverage across tissue samples, scRNA-seq enables identification of rare pathogenic cell populations and cell-state transitions that would otherwise remain obscured within averaged gene-expression profiles. However, preparation of tissues for scRNA-seq requires enzymatic and mechanical dissociation of lung samples into single-cell suspensions, a process that may alter stress-sensitive transcriptional programs, damage fragile cell populations, preferentially underrepresent structurally embedded vascular cells, and disrupt native cell–cell interactions that are central to pulmonary vascular remodeling. In addition, sparse transcript capture, batch effects, and substantial computational demands complicate quantitative comparisons across studies, while limited availability of high-quality PAH lung tissue remains a major practical constraint. Consequently, although scRNA-seq provides unprecedented cellular resolution, it incompletely captures the spatial and multicellular architecture of the diseased pulmonary vasculature when used in isolation.

Spatial transcriptomics extends scRNA-seq by preserving the anatomical context of gene expression within tissue sections. Ref. [84] applied spatial transcriptomics in familial PAH harboring a novel *KDR* mutation, revealing spatially restricted transcriptional programs of angiogenic dysregulation localized to plexiform lesions and remodeled small pulmonary arteries. This approach demonstrated that *KDR* mutation disrupts endothelial quiescence specifically within remodeled vascular compartments, providing mechanistic resolution that bulk or single-cell dissociation approaches cannot offer. By maintaining tissue architecture and spatial relationships between endothelial, immune, and mesenchymal cell populations, spatial transcriptomics enables interrogation of how pathogenic signaling networks are organized within remodeled vascular lesions. Nevertheless, current spatial transcriptomic platforms remain constrained by lower transcript depth, reduced single-cell resolution in some implementations, high analytical complexity, and substantial financial cost relative to conventional RNA sequencing approaches. As a result, spatial methods are currently less scalable for large cohort studies than bulk or single-cell transcriptomics. Genotype-stratified spatial transcriptomic studies will be essential for establishing whether distinct PAH gene mutations, such as *BMPR2* versus *SOX17* versus *TBX4*, produce convergent or divergent spatial vascular remodeling programs [84]. Collectively, bulk RNA sequencing, scRNA-seq, and spatial transcriptomics are increasingly viewed as complementary rather than competing technologies, with bulk approaches providing broad transcriptional coverage, scRNA-seq resolving cellular heterogeneity, and spatial transcriptomics restoring the anatomical context required to understand how disease-associated cell states interact within the pulmonary vascular microenvironment.

### 4.4. Epigenomics and DNA Methylation Profiling

Genome-wide DNA methylation profiling has established heritable and environmentally acquired epigenetic dysregulation as a central feature of PAH pathogenesis. Using whole-genome bisulfite sequencing and methylation arrays, ref. [98] demonstrated a global DNA hypermethylation signature in peripheral blood mononuclear cells from *TET2*-mutant PAH patients, with differentially methylated regions enriched at promoters of inflammatory response and vascular homeostasis genes. Ref. [97] extended these findings by correlating the *TET2* hypermethylation signature with specific T-cell phenotypes, further supporting the contribution of epigenetically mediated immune dysregulation to pulmonary vascular disease. Unlike genomic sequencing approaches, which identify relatively stable inherited variants, epigenomic profiling captures dynamic regulatory alterations shaped by both genetic susceptibility and environmental exposures, providing a potential mechanistic link between inherited predisposition, inflammation, hypoxia, and vascular remodeling.

Chromatin accessibility profiling using ATAC-seq in PAH patient-derived pulmonary artery endothelial cells has revealed disease-specific open chromatin at regulatory elements governing *KLF4*-dependent vasoprotective gene programs [52], and *FOXF1*-bound regulatory elements linked to DNA repair capacity [54]. The integration of epigenomic maps with GWAS data can prioritize functional variants disrupting transcription factor binding at disease-relevant regulatory regions, thereby bridging statistical associations to molecular mechanisms. This integrative approach is particularly important for the interpretation of non-coding GWAS loci, where epigenomic annotation can identify disease-relevant enhancers and regulatory elements that would otherwise remain biologically unresolved.

However, interpretation of epigenomic data in PAH remains challenging because many observed methylation and chromatin accessibility changes may represent secondary consequences of inflammation, hypoxia, vascular remodeling, aging, or pharmacological treatment rather than primary pathogenic drivers. Furthermore, epigenetic signatures are highly cell-type specific, and analyses performed using bulk peripheral blood or mixed lung tissue samples may obscure critical regulatory programs operating within rare endothelial or immune cell populations. Technical variability between methylation platforms and the temporal instability of epigenetic modifications also complicate reproducibility across cohorts. Consequently, integration with single-cell transcriptomics, chromatin accessibility profiling, and functional genomic approaches will be essential for distinguishing causal epigenetic mechanisms from downstream disease-associated regulatory alterations. Collectively, epigenomic technologies provide a critical intermediary layer linking inherited genetic variation to altered transcriptional states, but their greatest value emerges when interpreted alongside genomic, transcriptomic, and functional datasets within a broader multi-omics framework.

### 4.5. Proteomics and Metabolomics

High-throughput plasma proteomics using aptamer-based affinity platforms has identified circulating protein signatures that discriminate PAH from other forms of pulmonary hypertension. Of particular note, plasma BMP9 (encoded by *GDF2*) and BMP10 serve as quantitative biomarkers of BMP pathway activity: ref. [40] demonstrated that PAH patients with *GDF2* mutations have significantly reduced plasma BMP9, confirming that ligand insufficiency is directly measurable in the circulation. Compared with genomic approaches, which primarily identify inherited susceptibility, proteomics provides a dynamic functional readout of downstream pathway activity and disease state, thereby offering potential utility for disease monitoring, prognostication, and therapeutic stratification. Integration of proteomics with genetic variant data via protein quantitative trait loci (pQTL) analyses further enables causal inference, distinguishing genetically regulated proteins mechanistically linked to PAH risk from those merely co-expressed with disease. This represents an important advance over conventional biomarker studies, which often struggle to differentiate causal pathogenic mediators from secondary inflammatory or hemodynamic consequences of advanced disease.

Untargeted metabolomics of PAH patient plasma and lung tissue has consistently identified perturbations in glycolysis, the TCA cycle, fatty acid oxidation, and polyamine metabolism, reflecting the Warburg-like metabolic shift characteristic of hyperproliferative pulmonary vascular cells. Ref. [74] demonstrated that a single pathogenic *NFU1* mutation is sufficient to reprogram smooth muscle cellular metabolism from oxidative phosphorylation to aerobic glycolysis, establishing a direct genotype-to-metabolome link. Similarly, ref. [58] showed that *ATP13A3* variants disrupt polyamine transport and intracellular polyamine homeostasis in pulmonary artery endothelial cells, producing a distinct metabolic vulnerability detectable by metabolomics. These genotype-specific metabolic signatures represent both mechanistic evidence and potential biomarkers for disease monitoring and therapeutic stratification, illustrating how metabolomics can bridge inherited genetic defects to downstream cellular phenotypes.

However, despite their translational promise, both proteomic and metabolomic profiles are highly sensitive to comorbidities, medications, diet, tissue handling, circadian variation, and analytical platform differences, which can complicate reproducibility across independent cohorts. Furthermore, many observed metabolic and proteomic alterations may reflect downstream adaptive responses to chronic vascular remodeling, hypoxia, or right heart dysfunction rather than primary pathogenic drivers. In contrast to genomic variants, which are relatively stable throughout life, proteomic and metabolomic states are dynamic and context dependent, making longitudinal interpretation more complex. Consequently, the biological relevance of candidate biomarkers often requires validation through integration with genomic, transcriptomic, and functional datasets. Collectively, proteomics and metabolomics provide an essential functional dimension to PAH systems biology by capturing the downstream biochemical consequences of genetic and epigenetic dysregulation, but their greatest mechanistic value emerges when interpreted alongside complementary multi-omics and experimental approaches.

### 4.6. iPSC-Derived Models and CRISPR Functional Genomics

Patient-derived induced pluripotent stem cells (iPSCs) reprogrammed into pulmonary artery endothelial or smooth muscle cells provide a human genetic platform for dissecting the cellular consequences of PAH-associated variants in native cellular backgrounds. Unlike genomic and transcriptomic approaches, which primarily identify disease-associated variants or molecular signatures, iPSC-derived models enable direct experimental interrogation of variant function within disease-relevant human vascular cell types. iPSC-derived pulmonary artery endothelial cells (PAECs) from patients with BMPR2 mutations recapitulate reduced BMP signaling, increased apoptosis susceptibility, and metabolic reprogramming toward glycolysis [114], while iPSC models of *SOX17* deficiency demonstrate β-catenin-driven hyperproliferation and impaired endothelial identity maintenance [49]. These systems therefore provide an important mechanistic bridge between genetic association and cellular phenotype, allowing functional validation of candidate disease genes identified through sequencing studies.

CRISPR-Cas9 genome editing applied to iPSC-derived endothelial cells further allows isogenic comparison of wild-type and mutant genotypes, isolating the contribution of individual variants from confounding genetic background effects. Ref. [54] used patient-derived PAECs overexpressing *FOXF1* to demonstrate restoration of angiogenic capacity, *KDR* expression, and DNA repair gene activity, establishing a functional rescue paradigm applicable to other PAH transcription factor genes. The combination of iPSC modeling and CRISPR-mediated genome editing has therefore emerged as a powerful complementary framework for validating pathogenicity, interrogating gene regulatory networks, and identifying potential therapeutic targets.

Nevertheless, important limitations remain. iPSC-derived vascular cells frequently retain developmentally immature phenotypes and incompletely reproduce the complex multicellular and hemodynamic environment of the pulmonary circulation. Critical features of PAH pathobiology, including endothelial–smooth muscle interactions, inflammatory cell recruitment, extracellular matrix remodeling, and chronic shear stress exposure, are difficult to fully model in conventional two-dimensional culture systems. In addition, prolonged culture, clonal variability, and epigenetic drift may introduce experimental heterogeneity, while CRISPR-based approaches remain susceptible to off-target editing effects and may oversimplify the polygenic interactions underlying disease penetrance and expressivity. Consequently, iPSC and genome-editing technologies are most informative when integrated with animal models, patient tissue analyses, and multi-omics profiling. Collectively, these approaches provide a crucial experimental platform for translating genetic discoveries into mechanistic insight, bridging the gap between variant identification and functional understanding of pulmonary vascular disease.

### 4.7. Integrative Multi-Omics and Artificial Intelligence

The integration of genomic, transcriptomic, epigenomic, proteomic, and metabolomic datasets using frameworks such as multi-omics factor analysis (MOFA) and weighted gene co-expression network analysis (WGCNA) enables the identification of molecular programs coordinately dysregulated across omics layers. Unlike single-platform approaches, which capture only isolated dimensions of disease biology, integrative multi-omics frameworks provide systems-level insight into how inherited genetic variants propagate through regulatory, transcriptional, and metabolic networks to produce pulmonary vascular remodeling. These integrative analyses have demonstrated that diverse genetic lesions, spanning *BMPR2* receptor haploinsufficiency, *SOX17* transcriptional dysregulation, *TET2* epigenetic reprogramming, and *ATP13A3* metabolic vulnerability, converge on shared downstream molecular programs of endothelial inflammatory activation, BMP pathway suppression, and metabolic reprogramming [4]. Importantly, this convergence suggests that apparently distinct genetic subtypes of PAH may share common downstream pathogenic pathways, thereby providing a rationale for pathway-directed rather than purely gene-specific therapeutic strategies.

Artificial intelligence and machine learning approaches are increasingly being deployed to leverage multi-omics PAH datasets for risk prediction, disease progression modeling, and treatment response stratification at the individual patient level. Graph neural networks applied to multi-omics interaction networks hold the potential to identify non-obvious genetic modifiers and gene–gene interactions contributing to PAH penetrance and expressivity. These computational approaches are particularly valuable for analyzing the high dimensionality and network complexity generated by integrated omics datasets, which often exceed the interpretive capacity of conventional statistical frameworks.

However, despite their conceptual power, multi-omics and AI-driven approaches remain constrained by several important limitations. Most PAH cohorts remain relatively small because of the rarity of the disease, increasing susceptibility to overfitting, and limiting statistical generalizability across populations. Heterogeneity in tissue sources, sequencing platforms, clinical phenotyping, and bioinformatic pipelines further complicate cross-study integration and reproducibility. In addition, many machine learning models function as “black box” systems with limited interpretability, raising concerns regarding biological plausibility, transparency, and clinical applicability. The integration of multiple omics layers also substantially increases computational demands and analytical complexity, while distinguishing causal molecular drivers from secondary downstream alterations remains a major unresolved challenge. Consequently, robust multicenter datasets, standardized analytical frameworks, and experimental validation using functional genomic models will be essential before precision multi-omics approaches can be reliably translated into routine clinical practice.

Taken together, the convergence of next-generation sequencing, multi-omics integration, patient-derived cellular models, and AI-powered analytics is transitioning PAH research from single-gene discovery toward a comprehensive systems understanding of a genetically complex disease. Importantly, no single technology independently captures the full biological complexity of PAH; rather, the major advances in defining PAH genetic architecture have emerged through the convergence of complementary platforms spanning variant discovery, regulatory mapping, cellular resolution, functional validation, and systems-level computational integration.

## 5. Clinical Translation and Implications for Practice

The molecular-genetic discoveries reviewed in this article have direct and expanding implications for clinical practice across four principal domains: genetic diagnosis and cascade screening, risk stratification and prognostication, therapeutic decision-making, and genotype-guided intervention strategies.

### 5.1. Genetic Diagnosis and Cascade Screening

Genetic testing is now recommended for all patients with heritable PAH and idiopathic PAH by international guidelines, including the European Society of Cardiology/European Respiratory Society (ESC/ERS) and the American College of Chest Physicians [4,19]. Panel-based next-generation sequencing encompassing the 30 genes reviewed here, particularly the eight ClinGen-classified Definitive genes (*BMPR2*, *ACVRL1*, *ENG*, *GDF2*, *TBX4*, *SOX17*, *ATP13A3*, *EIF2AK4*), achieves a diagnostic yield of approximately 20–30% in idiopathic PAH and greater than 75% in familial PAH when combined with whole-genome sequencing approaches [4,75]. Identification of a pathogenic variant enables cascade genetic screening of first-degree relatives, facilitating pre-symptomatic diagnosis in mutation carriers before hemodynamic compromise develops. Early identification of at-risk individuals enables enhanced surveillance, lifestyle modification, and earlier initiation of therapy. However, the incomplete penetrance of most PAH-associated variants, particularly *BMPR2* (~20–30%), *ENG*, and *ACVRL1*, requires that genetic testing results be interpreted within a multidisciplinary framework that integrates clinical, physiological, and imaging data, rather than used in isolation to assign disease risk [19,20].

Critically, despite the increasing accessibility of next-generation sequencing, important limitations remain in the clinical implementation of genetic screening for PAH. A substantial proportion of patients with strong clinical phenotypes still lack identifiable pathogenic variants, highlighting the likelihood that additional undiscovered genes, non-coding regulatory variants, epigenetic mechanisms, and gene–environment interactions contribute to disease pathogenesis. Furthermore, many detected variants are classified as variants of uncertain significance (VUS), creating challenges in clinical interpretation and genetic counseling. This limitation is particularly relevant in underrepresented populations, where limited reference genomic databases may increase the risk of variant misclassification and reduce diagnostic accuracy. Consequently, the expanding use of broad sequencing panels may improve sensitivity but can simultaneously increase interpretative complexity without necessarily improving clinical prognostication.

Another important consideration is that the presence of a pathogenic mutation does not reliably predict disease severity, age of onset, or therapeutic response. Even among carriers of the same *BMPR2* mutation, marked phenotypic heterogeneity is observed, suggesting that secondary genetic modifiers, sex hormones, inflammation, metabolic dysregulation, and environmental exposures substantially influence disease penetrance and progression. This variability complicates risk stratification and raises important ethical and psychological concerns during cascade screening, particularly in asymptomatic relatives who may never develop clinically significant PAH. Therefore, while genetic diagnosis represents a major advance toward precision medicine in PAH, its current utility remains more effective for identifying susceptibility and guiding surveillance than for accurately predicting individual disease trajectory or tailoring treatment strategies.

### 5.2. Risk Stratification and Prognostication

Genotype is an established prognostic modifier in PAH. Patients with *BMPR2* mutations present at a younger age, exhibit more severe hemodynamic impairment at diagnosis (higher mean pulmonary artery pressure and pulmonary vascular resistance, lower cardiac output), and demonstrate reduced survival compared with mutation-negative patients, even after adjustment for disease severity [19,20]. The *BMPR2* mutation-associated survival disadvantage is independent of treatment and reflects fundamentally altered vascular biology rather than differential treatment access. Similarly, *ACVRL1* and *ENG* mutations in the context of HHT-associated PAH are associated with earlier onset and potentially more aggressive vascular disease, requiring heightened monitoring [30]. Conversely, mutations in genes with lower penetrance or milder signaling defects may be associated with later disease onset and a more indolent course. Genotype therefore provides an additional dimension to established risk stratification tools such as the REVEAL 2.0 score and the ESC/ERS low/intermediate/high-risk model, and its systematic incorporation into prognostic algorithms represents a priority for future clinical guideline development.

Nevertheless, the prognostic integration of genotype into routine clinical practice remains incompletely developed and faces several important limitations. Although *BMPR2* mutations consistently correlate with poorer outcomes at the population level, substantial heterogeneity exists among individual carriers, limiting the predictive precision of genotype alone. Patients harboring identical pathogenic variants may exhibit markedly different disease trajectories, ranging from asymptomatic lifelong carriers to rapidly progressive right heart failure. This variability indicates that additional modifiers, including epigenetic regulation, inflammatory signaling, sex-specific hormonal influences, metabolic dysfunction, and environmental exposures, significantly shape clinical phenotype beyond the primary mutation itself. Consequently, reliance on genotype without concurrent physiological and hemodynamic assessment may oversimplify the biological complexity of PAH progression.

Furthermore, most currently available prognostic models, including REVEAL 2.0 and ESC/ERS risk stratification systems, were primarily developed using clinical, exercise, imaging, and hemodynamic parameters rather than comprehensive molecular datasets. While the incorporation of genetic information has strong theoretical value, robust evidence demonstrating that genotype-guided risk prediction improves long-term clinical outcomes remains limited. In addition, the rarity of several PAH-associated mutations restricts the availability of sufficiently powered longitudinal studies needed to define mutation-specific prognostic trajectories. This limitation is particularly relevant for recently identified genes such as *ATP13A3* and *SOX17*, where long-term natural history data remain incomplete. Therefore, although precision-risk stratification based on genotype represents an important future direction, current evidence supports its role primarily as an adjunctive rather than standalone prognostic tool.

### 5.3. Therapeutic Decision-Making and Genotype-Guided Intervention

The convergence of diverse PAH genetic subtypes on impaired BMP signaling amplitude provides the biological rationale for pathway-directed therapeutics that confer benefit across genotypes. Sotatercept, a fusion protein that acts as an activin ligand trap to restore BMP/TGF-beta balance, received FDA and EMA approval in 2024 for PAH treatment based on the STELLAR trial demonstrating improved functional capacity and clinical outcomes [1]. Its mechanism of action directly targets the BMP signaling deficiency that is the unifying pathogenic theme across *BMPR2*-, *ACVRL1*-, *ENG*-, *GDF2*-, and *SMAD*-related PAH, suggesting that its benefits may be particularly relevant in genetically defined BMP-deficient disease subtypes, although prospective genotype-stratified evidence is still accumulating.

Beyond sotatercept, several genotype-specific therapeutic opportunities are emerging. Patients with *KCNK3* loss-of-function variants may theoretically benefit from pharmacological restoration of TASK-1 channel function; preclinical evidence supports the use of TASK-1 activators in rodent PAH models [66]. Patients with *EIF2AK4* mutations causing PVOD/PCH, a particularly aggressive and rapidly progressive form of disease, should be referred early for lung transplant assessment given the absence of effective medical therapies and very poor prognosis with conventional vasodilator treatment alone [70,71,72]. *BMPR2* mutation carriers who test positive on acute vasoreactivity testing should receive long-term calcium channel blocker therapy in accordance with current guidelines, a benefit not observed in non-carriers. For *TBX4*-associated pediatric PAH, recognition of the distinct developmental pathogenesis suggests that treatment protocols optimized for adult *BMPR2*-driven disease may require modification, and pediatric centers should apply *TBX4*-informed management strategies. In *TET2*-associated PAH, preliminary evidence implicating IL-1beta-driven inflammation suggests that anti-inflammatory strategies such as IL-1 receptor antagonism may represent a genotype-specific therapeutic avenue, although clinical validation is required [98,99].

Plasma BMP9 (encoded by GDF2) and BMP10 are emerging as pharmacodynamic biomarkers of BMP pathway activity that can be measured non-invasively and may facilitate therapeutic monitoring of sotatercept and other BMP-pathway-targeting agents [40]. Integration of genotype, plasma BMP ligand levels, and hemodynamic parameters could constitute a precision medicine framework for treatment selection and response monitoring in genetically defined PAH subgroups. The field is moving toward genotype-stratified clinical trial design, in which patients are enrolled and randomized based on underlying molecular subtype to optimize treatment matching and enhance sensitivity to detect genotype-specific therapeutic benefit.

## 6. Conclusions and Future Perspectives

The past two decades have established PAH as one of the most genetically complex rare diseases in medicine, with 30 genes now identified across mechanistically diverse functional categories: TGF-β/BMP signaling receptors (*BMPR2*, *ACVRL1*, *ENG*, *BMPR1B*), circulating BMP ligands (*GDF2*, *BMP10*), transcription factors (*TBX4*, *SOX17*, *KLF4*, *FOXF1*, *SMAD1*, *SMAD4*, *SMAD9*), membrane and polyamine transporters (*ATP13A3*, *AQP1*), potassium channel regulators (*KCNA5*, *KCNK3*, *ABCC8*), metabolic and mitochondrial genes (*EIF2AK4*, *NFU1*, *GGCX*), signaling receptors (*NOTCH3*, *KDR*), structural membrane proteins (*CAV1*, *PLEKHH2*), vasoactive regulators (*KLK1*), extracellular matrix mediators (*CBLN2*, *CD248*), and epigenetic regulators (*TET2*, *TOPBP1*). Despite this breadth, *BMPR2* remains the dominant genetic contributor, accounting for 53–86% of heritable PAH, while the remaining 29 genes each account for less than 5% of cases. This uneven distribution underscores that PAH genetics is characterized by one high-prevalence gene operating within a broad landscape of rare and ultra-rare contributors.

A unifying theme across this genetic landscape is incomplete penetrance and variable expressivity, which are most clearly documented for *BMPR2* (approximately 20–30% penetrance) but likely extend to most PAH risk genes. This incomplete penetrance provides the biological rationale for the two-hit hypothesis: germline mutations create a molecularly sensitized vascular state, but overt disease requires additional insults—chronic hypoxia, inflammation, hormonal influences, hemodynamic stress, or secondary genetic modifiers such as SNPs in *TGF-β1*, *HIF1A*, *SIRT3*, and *UCP2*—to trigger the transition from subclinical endothelial dysfunction to progressive vascular remodeling. Understanding the molecular basis of this threshold effect remains one of the most important unresolved questions in PAH biology, with direct implications for identifying which mutation carriers are at highest risk of disease progression.

A second unifying theme is convergence: genetically diverse PAH risk genes, operating through distinct upstream mechanisms—whether BMP ligand insufficiency, receptor haploinsufficiency, transcriptional dysregulation, ion channel failure, metabolic stress, or epigenetic reprogramming—converge on shared downstream phenotypes of endothelial dysfunction, smooth muscle proliferation, apoptosis resistance, and inflammatory vascular remodeling. This convergence has critical therapeutic implications: it suggests that targeting downstream nodes of pathway convergence, such as restoring BMP signaling amplitude (as achieved by sotatercept, approved in 2024 for PAH treatment), may confer benefit across multiple genetic subtypes rather than requiring genotype-specific interventions. At the same time, the mechanistic heterogeneity across PAH genes highlights opportunities for precision medicine, particularly in genetically defined subgroups where specific pathway defects may be pharmacologically tractable.

The integration of emerging technologies, whole-genome sequencing, single-cell and spatial transcriptomics, multi-omics data integration, iPSC-derived vascular models, and artificial intelligence is now enabling a systems-level understanding of how individual genetic variants propagate their effects through molecular and cellular networks to produce pulmonary vascular disease. These approaches are beginning to resolve the cell-type specificity of gene action, the temporal sequence of pathological events, and the modifying influence of epigenetic and environmental factors, thereby filling fundamental mechanistic gaps that patient cohort sequencing alone cannot address.

Several critical priorities remain for the field. First, large-scale international sequencing collaborations across ancestrally diverse populations are urgently needed to increase statistical power for rare variant discovery, improve genotype–phenotype correlations, and assess whether current genetic knowledge, derived predominantly from European cohorts, generalizes to global populations. Second, systematic functional validation of variants in genes with limited evidence (*BMPR1B*, *GGCX*, *NFU1*, *PLEKHH2*, *CD248*, *TOPBP1*) is essential to establish ClinGen-level evidence and enable their incorporation into clinical genetic testing panels. Third, the molecular basis of incomplete penetrance requires direct investigation through longitudinal studies integrating genetics, epigenomics, and environmental exposure data in mutation carriers who remain disease-free. Fourth, genetic findings must be more systematically translated into clinical practice, including standardized cascade screening protocols, genotype-informed prognostication, and genotype-stratified clinical trial design.

In summary, the genetic architecture of PAH has been transformed from a single-gene Mendelian disorder to a molecularly heterogeneous condition with contributions across 30 genes and multiple variant classes. The field is at an inflection point: the core genetic landscape is substantially defined, the mechanistic principles of BMP pathway disruption, endothelial vulnerability, and second-hit requirements are established, and the tools for systems-level investigation are now available. The central challenge is to translate this accumulated genetic and mechanistic knowledge into improved diagnostic precision, biologically informed prognostication, and most importantly, novel therapeutic strategies capable of halting or reversing pulmonary vascular remodeling in patients across the full spectrum of PAH genetic subtypes.

## Data Availability

No new data were created or analyzed in this study. Data sharing is not applicable to this article.

## Abbreviations

*ABCC8*	ATP-binding cassette subfamily C member 8
*ACVRL1*	Activin Receptor Type 1
*AQP1*	Aquaporin 1
*ATP13A3*	ATPase 13A3
BMP	Bone Morphogenetic Protein
*BMP10*	Bone Morphogenetic Protein 10
*BMPR1B*	Bone Morphogenetic Protein Receptor Type 1B
*BMPR2*	Bone Morphogenetic Protein Receptor 2
*CAV-1*	Caveolin-1
*CBLN2*	Cerebellin 2 precursor
*CD248*	Cluster of Differentiation 248
*EIF2AK4*	Eukaryotic Translation Initiation Factor 2 Alpha Kinase 4
*ENG*	Endoglin
*FOXF1*	FOXhead box protein F1
GCN2	General Control Nonderepressable 2
*GDF2*	Growth Differentiation Factor 2
*GGCX*	Gamma-Glutamyl Carboxylase
HPAH	Hereditary Pulmonary Arterial Hypertension
IPAH	Idiopathic Pulmonary Arterial Hypertension
*KCNA5*	Potassium voltage-gated channel subfamily A member 5
*KCNK3*	Potassium Channel Subfamily K Member 3
*KDR*	Kinase Insert Domain Receptor
*KLF4*	Kruppel-like factor 4
*KLK1*	Kallikrein-1
*NFU1*	*NFU1* Iron–Sulfur cluster scaffold homolog
*NOTCH3*	Neurogenic locus notch homolog protein 3
PAH	Pulmonary Arterial Hypertension
*PAI-1*	Plasminogen Activator Inhibitor-1
PH	Pulmonary Hypertension
*PLEKHH2*	Pleckstrin homology domain-containing, family H (with MyTH4 domain), member 2
SMAD	Mothers against decapentaplegic homolog
*SOX17*	SRY-BOX Transcription Factor 17
*TBX4*	T-box transcription factor 4
*TET2*	Tet methylcytosine dioxygenase 2
TGF-β	Transforming Growth Factor Beta
